# Effectiveness and Safety of Contemporary Drug Regimens for Multidrug- and Extensively Drug-resistant Tuberculosis in Children and Adolescents: A Global Systematic Review and Meta-analysis

**DOI:** 10.1097/INF.0000000000005269

**Published:** 2026-05-21

**Authors:** Wamaka Kaminyoge, Reuben Simfukwe, Mwiza Singini, Norman Sitali, Felix P. Chilunga

**Affiliations:** From the * Public Health Programme, Warwick Medical School, University of Warwick, Coventry, United Kingdom; † Clinical Research Education and Management Services (CREAMS), Queen Elizabeth Central Hospital, Blantyre, Malawi; ‡ South African Medical Research Council, Cape Town, South Africa; § Doctors Without Borders Germany, Berlin, Germany; ¶ Department of Public and Occupational Health, Amsterdam University Medical Centers, University of Amsterdam, Amsterdam, The Netherlands.

**Keywords:** multidrug-resistant tuberculosis, extensively drug-resistant tuberculosis, pediatric, contemporary regimens, meta-analysis

## Abstract

**Background:**

Children and adolescents with multidrug-resistant (MDR) or extensively drug-resistant (XDR) tuberculosis (TB) face major treatment challenges. Although newer all-oral regimens have transformed adult TB care, their use in younger populations has largely been extrapolated from adult studies, despite important biologic and clinical differences. Systematic and up-to-date evidence on the effectiveness and safety of contemporary regimens in children and adolescents is lacking. We conducted a global systematic review and meta-analysis to address this gap.

**Methods:**

We systematically searched PubMed, EMBASE, Web of Science, Cochrane Library and Google Scholar for studies published between January 1, 2015, and September 1, 2025. Eligible studies included children 0–19 years old with confirmed or presumed MDR/XDR-TB or rifampicin-TB receiving pharmacologic treatment. Outcomes were pooled using a random-effects meta-analysis. The primary outcome was treatment success (cure or completion); severe (grade 3/4) adverse events were assessed as secondary outcomes.

**Results:**

We included 14 studies comprising 490 children across 13 countries. Overall pooled treatment success was 89% (95% confidence interval: 84%–93%; *I*^2^ = 51.9%). Newer all-oral regimens achieved slightly higher success (91%) than injectable-containing regimens (83%). Severe grade 3/4 adverse event were sparse (7 of 14 studies); events occurred in 21% (95% confidence interval: 13%–33%), most commonly QT prolongation and anemia, with limited specific drug attribution.

**Conclusions:**

Newer, all-oral bedaquiline- and delamanid-based regimens for pediatric MDR/XDR-TB are effective and generally well tolerated, though the full safety profile remains incompletely characterized. In pediatric MDR/XDR-TB, where studies are rare, our findings provide the best available evidence and a foundation for improved treatment guidelines and future research.

Despite sustained global efforts to control tuberculosis (TB), it remains one of the leading infectious causes of death worldwide.^1,2^ The persistent burden of TB-related morbidity and mortality underscores the urgent need for more effective and tailored interventions.^1,2^ This challenge is further compounded by the emergence and global spread of drug-resistant forms of TB—namely multidrug-resistant TB (MDR-TB) and extensively drug-resistant TB (XDR-TB)—which threaten to derail decades of progress toward global TB elimination.^3,4^ According to the World Health Organization (WHO), MDR-TB is caused by Mycobacterium tuberculosis strains resistant to at least isoniazid and rifampicin, while XDR-TB refers to MDR-TB with additional resistance to any fluoroquinolone and at least 1 Group A drug (bedaquiline or linezolid).^5^

Managing MDR/XDR-TB is particularly complex due to prolonged, toxic and costly treatment regimens.^6^ These regimens impose a heavy burden on patients, caregivers, healthcare systems and national TB control programs.^7^ Treatment outcomes for MDR/XDR-TB are often suboptimal, with high rates of treatment failure, relapse and severe adverse events such as hepatotoxicity, nephrotoxicity and ototoxicity.^8^ The situation is especially dire in low- and middle-income countries, where access to advanced diagnostics and newer therapeutics remains limited. Reflecting this disproportionate burden, the WHO prioritizes the 30 most affected countries through its High-Burden Country lists to guide global TB control efforts, resource allocation, surveillance and research.^9^ These lists encompass MDR/rifampicin (RR)-resistant TB (MDR/RR-TB), where RR-resistant TB (RR-TB), defined by resistance to RR regardless of resistance to other drugs, is classified and treated within the same framework as MDR-TB and is routinely reported alongside it in the literature without disaggregation by resistance subtype.^9,10^

Children and adolescents face additional and unique vulnerabilities in the context of MDR/XDR-TB.^11^ They often present with nonspecific symptoms, possess developing immune systems, and are at heightened risk of rapid progression from TB infection to TB disease.^11,12^ In high-burden settings, household exposure is a major driver of primary drug-resistant TB infection among younger age groups.^13,14^ Beyond clinical implications, MDR/XDR-TB in children and adolescents disrupts education, affects mental health, places substantial financial strain on families and contributes to ongoing community transmission.^15^

Over the last decade, treatment for MDR/XDR-TB has evolved significantly with the introduction of newer, shorter and safer all-oral regimens, notably those containing bedaquiline and delamanid.^16,17^ However, the supporting evidence for these regimens remains largely derived from adult studies.^18^ Data specific to children and adolescents—on pharmacokinetics, safety, tolerability and optimal dosing—are still limited.^18^ As a result, treatment guidelines for children and adolescents often rely on indirect evidence, potentially overlooking important age-related physiologic and metabolic considerations.^18–20^

Two previous systematic reviews and meta-analyses have assessed treatment outcomes in children and adolescents with XDR- and pre-XDR-TB, reporting pooled success rates between 65% and 89%^10,21^ However, the first primarily evaluated older, injectable-based regimens, while the second—conducted in 2020—included only a small proportion (7%) of patients treated with newer all-oral regimens and was mainly based on studies from India and South Africa.^10,21^ Since then, a growing number of studies from a wider range of countries have examined the effectiveness of contemporary regimens in children and adolescents increasing the evidence base (over 5 years global new literature).^19,22^ However, there is still no up-to-date and comprehensive meta-analysis that has assessed the effectiveness and safety of contemporary all-oral regimens among children and adolescents.

Given the evolving therapeutic landscape and expanding global experience, there is a pressing need to synthesize the most current evidence on MDR/XDR-TB treatment in children and adolescents. We therefore conducted a systematic review and meta-analysis to assess the effectiveness of contemporary all-oral regimens in this younger population. Safety was also evaluated from the identified studies, with particular attention to grade 3 and 4 adverse events as classified by the Common Terminology Criteria for Adverse Events (CTCAE), which represent severe and life-threatening toxicities most likely to necessitate drug discontinuation or hospitalization, to enable an integrated assessment of the benefit–risk profile of these treatments. The findings aim to support the development of age-appropriate clinical guidelines, inform national and global policy initiatives, and align with priorities set by the WHO and the United Nations High-Level Meeting on TB.^16^

## METHODOLOGY

### Study Design and Registration

We conducted a systematic review and meta-analysis to evaluate the effectiveness of contemporary drug treatment options for MDR/XDR-TB in children and adolescents (0–19 years). Contemporary regimens were defined as newer all-oral combinations, containing either bedaquiline, delamanid or pretomanid. Injectable-containing regimens were the longer treatment courses of 18–24 months that often-included injectable agents such as kanamycin, amikacin or capreomycin, in combination with other second-line drugs. Both types of regimens were included if studied within the review window, enabling a comparative analysis between newer all-oral therapies and the injectable-containing regimens. The review followed the Preferred Reporting Items for Systematic Reviews and Meta-Analyses (2020) guidelines. The protocol was prospectively registered in the International Prospective Register of Systematic Reviews (registration number: CRD42024604224).

### Eligibility Criteria

We included studies involving children and adolescents 0–19 years of age with pulmonary and/or extrapulmonary MDR/XDR-TB. Studies that included patients with RR-TB were also eligible, as RR-TB is managed within the same treatment framework as MDR-TB and is routinely reported alongside MDR-TB in the literature without disaggregation.^9,10,23^ Both pulmonary and/or extrapulmonary disease forms were included to ensure comprehensive capture of pediatric MDR/XDR-TB, as most studies report mixed presentations and pulmonary TB remains the most common form. We included studies with microbiologically confirmed or presumed MDR/XDR-TB. Microbiologic confirmation was based on culture or molecular drug susceptibility testing. Presumed MDR/XDR-TB was defined as a clinical diagnosis based on prior TB treatment history, contact with a known drug-resistant TB case, or failure of first-line therapy despite documented good adherence. Presumed cases were included to account for limited diagnostic capacity in low-resource settings, where microbiologic confirmation is often unavailable. We included studies that assessed treatment effectiveness and/or safety and used eligible designs, including randomized controlled trials, nonrandomized trials, cohort studies, case–control studies or case series. We included only studies reporting pediatric-specific outcomes, including adolescents 15–19 years old. We excluded studies that focused exclusively on adults, evaluated only first-line TB regimens, did not report pulmonary TB outcomes or were not published in English.

### Information Sources and Search Strategy

We systematically searched 4r electronic databases—PubMed, EMBASE, Web of Science and the Cochrane Library—for studies published from January 1, 2015, to September 1, 2025 (as newer all-oral regimens started appearing in the mid to late 2010s and to capture the most recent contemporary treatments). Gray literature was searched via Google Scholar.

The primary objective of the search was to identify studies reporting on the effectiveness of pharmacologic treatment regimens for MDR/XDR-TB in children and adolescents. Safety outcomes were extracted secondarily from these effectiveness studies to allow for an integrated assessment of the benefit–risk profile. Accordingly, the search strategy was primarily structured around treatment effectiveness, with safety data collected where available from the same set of studies.

The search strategy combined Medical Subject Headings (MeSH) and free-text keywords related to tuberculosis, drug resistance, pediatric populations and pharmacologic treatment. The following MeSH terms were used: “Tuberculosis, Multidrug-Resistant” [MeSH], “Tuberculosis, Extensively Drug-Resistant” [MeSH], “Therapeutics” [MeSH], “Drug Therapy” [MeSH], “Child” [MeSH], “Infant” [MeSH], and “Adolescent” [MeSH]. Drug-specific MeSH terms included “Bedaquiline” [MeSH], “Delamanid” [MeSH], Pretomanid” [MeSH], “Linezolid” [MeSH], “Clofazimine” [MeSH] and “Fluoroquinolones” [MeSH].

We also included relevant free-text terms such as “tuberculosis,” “MDR-TB,” “XDR-TB,” “drug-resistant tuberculosis,” “treatment outcome,” “children,” “pediatric,” “paediatric,” “bedaquiline,” “delamanid,” “linezolid,” “pretomanid, “clofazimine,” “fluoroquinolones,” “capreomycin,” “amikacin,” “levofloxacin” and “moxifloxacin.” Older medications, including capreomycin, amikacin, levofloxacin and moxifloxacin, were included to ensure comprehensive coverage of all drug regimens for MDR/XDR-TB in children within the study window. This approach enables a comparative analysis between newer all-oral regimens and older injectable/oral drugs, providing context for improvements in treatment outcomes with newer therapies.

Boolean operators (AND, OR) were used to combine search terms, and filters were applied to limit results to human studies published in English. Reference lists of included articles were manually screened to identify any additional eligible studies. Adverse events were extracted only when studies reported effectiveness.

### Study Selection and Data Extraction

All retrieved records were imported into EndNote X9 for de-duplication. Two reviewers independently screened titles and abstracts and assessed full texts for eligibility and extracted relevant data into Microsoft Excel. Disagreements were resolved by discussion or through consultation with a third reviewer. Data extraction was performed using a standardized form and included study characteristics (author, year and location), clinical setting, participant demographics, diagnostic methods, treatment regimens, treatment duration, comorbidities and treatment outcomes. Resistance categories were not classified in this review, but were retained as reported in the original studies. Adverse events were extracted when reported as severe or classified as grade 3 or 4, based on the CTCAE.^24^ Severe or grade 3–4 adverse events were prioritized because they represent clinically significant toxicity, often require treatment modification or discontinuation, and are essential for assessing the benefit–risk profile of MDR/XDR-TB regimens in pediatric populations. Adverse effects reporting across studies was inconsistent, as some studies did not use a formal grading system. Treatment outcomes were defined according to the 2020 WHO guidelines.^23^ Favorable outcomes included cure or treatment completion, while unfavorable outcomes included treatment failure and death, and lost to follow-up.

### Quality Assessment

The methodologic quality of the included studies was assessed using validated tools. Observational studies were evaluated using the Newcastle–Ottawa Scale,^25^ which assesses quality across selection, comparability and outcome domains. Scores of 7–9 were classified as high quality. Randomized controlled trials were assessed using the Cochrane Risk of Bias Tool,^26^ which evaluates risk across multiple domains including sequence generation, allocation concealment, blinding, incomplete outcome data and selective reporting. Publication bias was assessed using funnel plots and Egger’s test. The overall certainty of evidence was assessed using the GRADE approach, which considers risk of bias, inconsistency, indirectness, imprecision and publication bias. Discrepancies were resolved through consensus or, when necessary, third-party adjudication.

### Data Synthesis and Statistical Analysis

A meta-analysis was conducted to estimate the overall pooled proportion of children and adolescents achieving favorable treatment outcomes during the study period, as well as the overall proportion experiencing severe (grade 3–4) adverse events. Individual serious adverse events were sparsely reported to be meta-analyzed separately or by drug attribution. Ninety-five percent confidence intervals (CIs) were calculated using the Wilson score interval method. A random-effects model (DerSimonian and Laird method) was applied to account for between-study heterogeneity. Heterogeneity was assessed using Cochran’s Q test (with *P* < 0.10 considered significant to allow detection of even moderate heterogeneity) and quantified using the *I*^2^ statistic. *I*^2^ values of 25%, 50% and 75% were interpreted as indicating low, moderate and high heterogeneity, respectively. All statistical analyses were performed in R version 4.4.2 using the *meta* and *metafor* packages.

### Subgroup and Sensitivity Analysis

Subgroup analyses were conducted by regimen type (all-oral vs. injectable-containing), inclusion of bedaquiline or delamanid (yes vs. no), regimen duration and type (3 groups: all-oral short (≤12 months), all-oral long (>12 months) and injectable-containing long (>12 months), as no injectable-containing short-duration studies existed in this dataset), country income level (to reflect resource settings) and TB burden (high vs. non-high, to capture epidemiologic context). Sensitivity analyses were performed by excluding studies with 100% success rates, which may inflate pooled estimates, and by restricting analyses to studies with a low risk of bias to reduce methodologic influence.

## RESULTS

### Study Selection

A total of 955 records were identified through systematic database searches: 905 from PubMed, EMBASE, Web of Science and the Cochrane Library, and an additional 50 from Google Scholar (Figure, Supplemental Digital Content 1, https://links.lww.com/INF/G621). After removal of 460 duplicates, 495 unique records were screened based on titles and abstracts, resulting in the exclusion of 439 studies. The remaining 56 full-text articles underwent detailed review, with 42 further excluded due to not meeting the inclusion criteria. Ultimately, 14 studies fulfilled all eligibility requirements and were included in the systematic review and meta-analysis (Figure, Supplemental Digital Content 1, https://links.lww.com/INF/G621).

### Study Characteristics

The 14 included studies were published between 2016 and 2024 and involved 490 children with MDR- or XDR-TB (Table 1; Table, Supplemental Digital Content 2, https://links.lww.com/INF/G622). The studies were conducted in diverse countries, including Belarus, Ukraine, South Africa, India, Tajikistan, Uzbekistan, Russia, Philippines, United States, Chile, China, Brazil and Afghanistan. Most studies used a retrospective cohort design, with 3 clinical trials and 2 observational studies. Across studies, diagnosis and resistance confirmation were established using culture and/or molecular tests (eg, Xpert Mycobacterium tuberculosis/rifampicin), line probe assays with study-specific diagnostic pathways summarized in Table, Supplemental Digital Content 2, https://links.lww.com/INF/G622. Comorbidities reported included HIV, undernutrition, anemia, epilepsy, diabetes and hepatitis. Treatment regimens frequently include bedaquiline and/or delamanid, often combined with linezolid, fluoroquinolones (Levofloxacin and Moxifloxacin), cycloserine, clofazimine and injectables (amikacin, capreomycin, kanamycin and streptomycin; Table, Supplemental Digital Content 2, https://links.lww.com/INF/G622).

**TABLE 1.** Characteristics of Studies Included in Analysis

No.	Author, Year, Study Country	Study Design	Age Range (yr)	Sample Size (Enrolled/Analyzed)	Resistance Category (Reported/Analyzed)	Drug Regimen	Injectable Used (Y/N)	Treatment Duration	Treatment Success (Outcomes Available)
1	Solodovnikova et al (2021), Belarus^20^	Retrospective cohort	10–17	40/38	RR/MDR-TB MDR-TB XDR-TB/ RR-MDR-TB XDR-TB	FQ, Bdq, Dlm, Lzd	N	Up to 18 mo	38/38 (100%)
2	Sakhelashvili et al (2023), Ukraine^27^	Retrospective cohort	0–17	67/67	MDR-TB XDR-TB/ MDR-TB XDR-TB	Bdq, Dlm, Lzd, Cfz, Cs	N	Up to 18–20 mo	40/40 (100%)
**3**	Mohr-Holland et al (2020), South Africa^28^	Retrospective cohort	10–19	22/22	RR-TB/ RR-TB	Bdq/ Dlm/ Bdq + Dlm	N	Up to 8.4 mo in BDQ treatment Up to 14.4 mo in DLM treatment	17/22 (77%)
4	Das et al (2020), India^29^	Retrospective cohort	3–18	24/24	Pre-XDR-TB XDR-TB/ Pre-XDR-TB XDR-TB	Bdq/ Dlm/ Bdq + Dlm (background regimen)	N	Up to 98 wk (24 mo)	23/24 (96%)
5	Achar et al (2017), South Africa, Tajikistan, and Uzbekistan^30^	Retrospective cohort	10–17	27/23	Pre-XDR-TB XDR-TB/ Pre-XDR-TB XDR-TB	Bdq, Lzd, Cfz, Mfx, Imi	N	6 mo	23/23 (100%)
**6**	Pirmahmadzoda et al (2021), Tajikistan	Retrospective cohort	0–17	60/60	RR/MDR-TB Pre-XDR-TB XDR-TB/ RR-MDR-TB Pre-XDR-TB XDR-TB	Bdq, Dlm, Cm/Am, Lfx/Mfx, Pto, Cs, PAS, Lzd, Cfz	N	Up to 36 months	58/60 (100%)
**7**	Ghosh et al (2022), Russia, India, South Africa^31^	Retrospective cohort	6–17	34/34	RR-TB MDR-TB Pre-XDR-TB XDR-TB/ Unclear	Dlm + background regimen	N	Up to 6 mo	20/25 (80%)
8	Hafkin et al (2018), South Africa, India, Chile, USA^32^	Retrospective cohort	8–17	6/6	MDR-TB Pre-XDR-TB XDR-TB/ Unclear	Bdq, Dlm (combined)	N	6 mo	6/6 (100%)
9	Moodliar et al (2021), Philippines, Russia, South Africa^33^	Clinical Trial	5–17	21/21 (11 cohort 1, 10 cohort 2, MGIT-evaluable patients)	MDR-TB RR-TB XDR-TB/MDR-TB	Bdq + background regimen	N	24 wk	9/11 (82%)
10	Garcia-Prats et al (2022), Philippines and South Africa^34^*	Clinical Trial	0–17	37/37	MDR-TB/MDR-TB	Dlm + optimized background regimen	N	Delamanid treatment for 6 mo	33/37 (89%)
11	Smirnova et al (2016), Russia^35^	Observational	0–<15 15–18	56/52	MDR-TB/ XDR-TB	Pto, PAS, Cs, EMB, kanamycin and/or Cm)	Y	Up to 24 mo	33/36 (92%) (0 to <15 age group) 14/16(88%) (15 to 18 age group) 47/52(90%) (All ages)
12	Da Costa et al (2023), Brazil^36^†	Observational	0–14	46/46	MDR-TB RR-TB XDR-TB/ MDR-TB RR-TB XDR-TB	Cm, EMB, PZA, Lfx, Trd	Y	Up to 21 mo	34/46(74%)
13	Sun et al (2024), China^22^	Retrospective cohort	10–17	79/79 (37 in the BDQ group and 42 patients in the matched control group)	RR-TB MDR-TB Pre-XDR-TB XDR-TB/ RR-MDR-TB	Bdq, FQ, Lzd, Cs, Cfz, PZA, Pto, PAS, EMB	N	Up to 18–20 mo (Bedaquiline was given for 24 wk)	74/79 (94%) BDQ group was 100% (37/37)
14	Mesic et al (2024), Afghanistan^19^	Clinical Trial	0–14	27/27	RR-TB Pre-XDR-TB/ RR-TB	Bdq, Dlm, Lfx, Lzd, Cfz, Z, Cs	N	9 mo	23/27 (85%)

* 64% of participants were under 5 years old

† 15% of participants were under 5 years old.

Am indicates amikacin; Bdq, bedaquiline; Cfz, clofazimine; Cm, capreomycin; Cs, cycloserine; Dlm, delamanid; EMB, ethambutol; FQ, fluoroquinolone; Imi, imipenem; Lfx, levofloxacin; Lzd, linezolid; Mfx, moxifloxacin; PAS, para-aminosalicylic acid; Pto, protionamide; PZA or Z, pyrazinamide; Trd, terizidone.

Differences between enrolled and analyzed participants reflect loss to follow-up, transfer out or missing outcome data as reported in the original studies.

Definition of cured or completed treatment is based on the World Health Organization (WHO) definitions of successful TB treatment outcomes. Complete-case analyses were performed using the final sample with documented treatment outcomes. For cured patients; at least three consecutive negative cultures, taken at least 30 days apart in the final 12 months of treatment. Completed treatment means the patient did not have lab confirmation but they must finish the full treatment course without evidence of failure.

Treatment durations ranged from 6 to 36 months (Table 1). Duration of regimen taking less than or equal to 12 months was categorized as short, while those taking more than 12 months as long. Regimens were classified as all-oral if no injectable agent was used and injectable-containing if an injectable agent was included. Across the included studies, 12 studies used all-oral regimen, and 2 studies used an injectable-containing regimen. Bedaquiline-containing regimen was reported in 10 studies and delamanid-containing regimen was reported in 9 studies (Table 1; Table, Supplemental Digital Content 2, https://links.lww.com/INF/G622).

### Treatment Success Proportions by Study

Individual study success proportions ranged from 74% to 100% (Table 1). Most studies reported success rates above 80%. The study from China (Sun et al) contributed the largest number of children successfully treated (74 of 79, 94%; Table 1). Several smaller studies reported 100% success rates, including those by Solodovnikova et al (Belarus), Sakhelashvili et al (Ukraine), Achar et al (South Africa, Tajikistan, Uzbekistan), Pirmahmadzoda et al (Tajikistan) and Hafkin et al (multinational). The lowest success rate (74%) was observed in the Brazilian study by Da Costa et al, where the burden of HIV and undernutrition was high.

### Overall Pooled Treatment Effectiveness

Among the 490 children analyzed, 445 achieved favorable outcomes. The overall pooled treatment success rate was 89% (95% CI: 84%–93%, Fig. 1). Heterogeneity across studies was moderate (*I*^2^ = 51.9%), with Cochran’s Q test indicating statistical significance (*P* < 0.10). This variability likely reflects differences in drug regimens, diagnostic criteria, healthcare systems and patient-level factors such as comorbidities.

**FIGURE 1.**
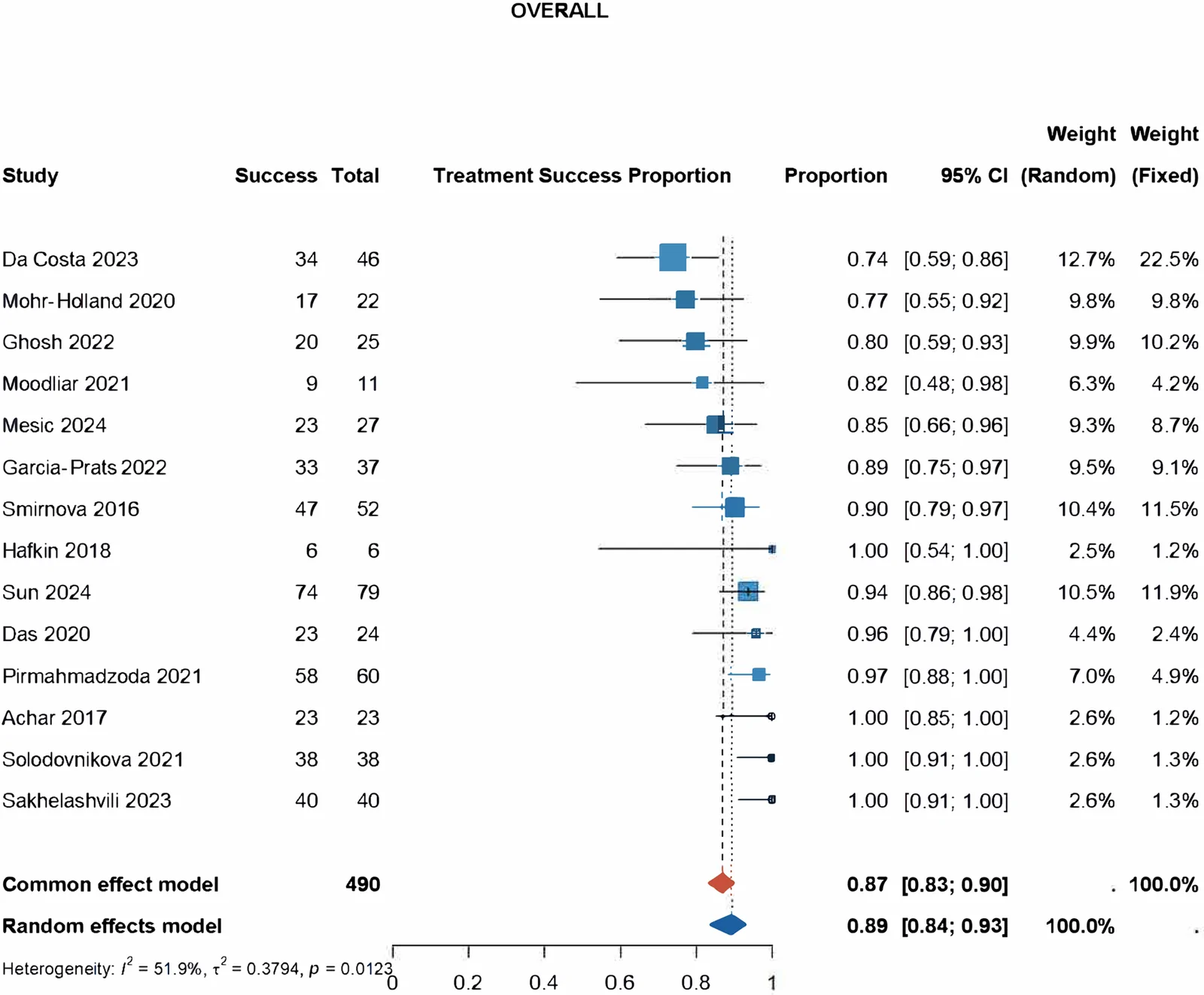
Forest plot illustrating overall pooled treatment success across all 14 included studies. CI indicates confidence interval.

### Subgroup Analyses on Pooled Effectiveness

Subgroup analyses are presented in Figure 2 and Figures, Supplemental Digital Content 3–7, https://links.lww.com/INF/G622. All-oral regimens achieved a slightly higher success rate (91%) than injectable-containing regimens (83%), though this difference was not statistically significant (*P* = 0.304). Similarly, bedaquiline-containing regimens showed marginally higher success (92%) than those without (84%), and delamanid-containing regimens performed comparably (90%) relative to delamanid-free regimens (88%); neither difference reached statistical significance (*P* = 0.062 and *P* = 0.637, respectively). Regarding duration, all-oral short regimens (≤12 months) achieved the highest success rate (96%), followed by all-oral long regimens (>12 months) at 84% and injectable-containing long regimens (>12 months) at 83%; the difference between all-oral short and all-oral long regimens was statistically significant (*P* = 0.002). Success rates were slightly higher in low-income settings (93%) than in upper- and middle-income settings (90%), and in non-high-burden countries (93%) compared with high-burden settings (90%); however, neither difference was statistically significant (*P* = 0.481 and *P* = 0.448, respectively). Across all subgroup analyses, moderate heterogeneity was observed (*I*^2^ = 51.9%, *P* = 0.0123).

**FIGURE 2.**
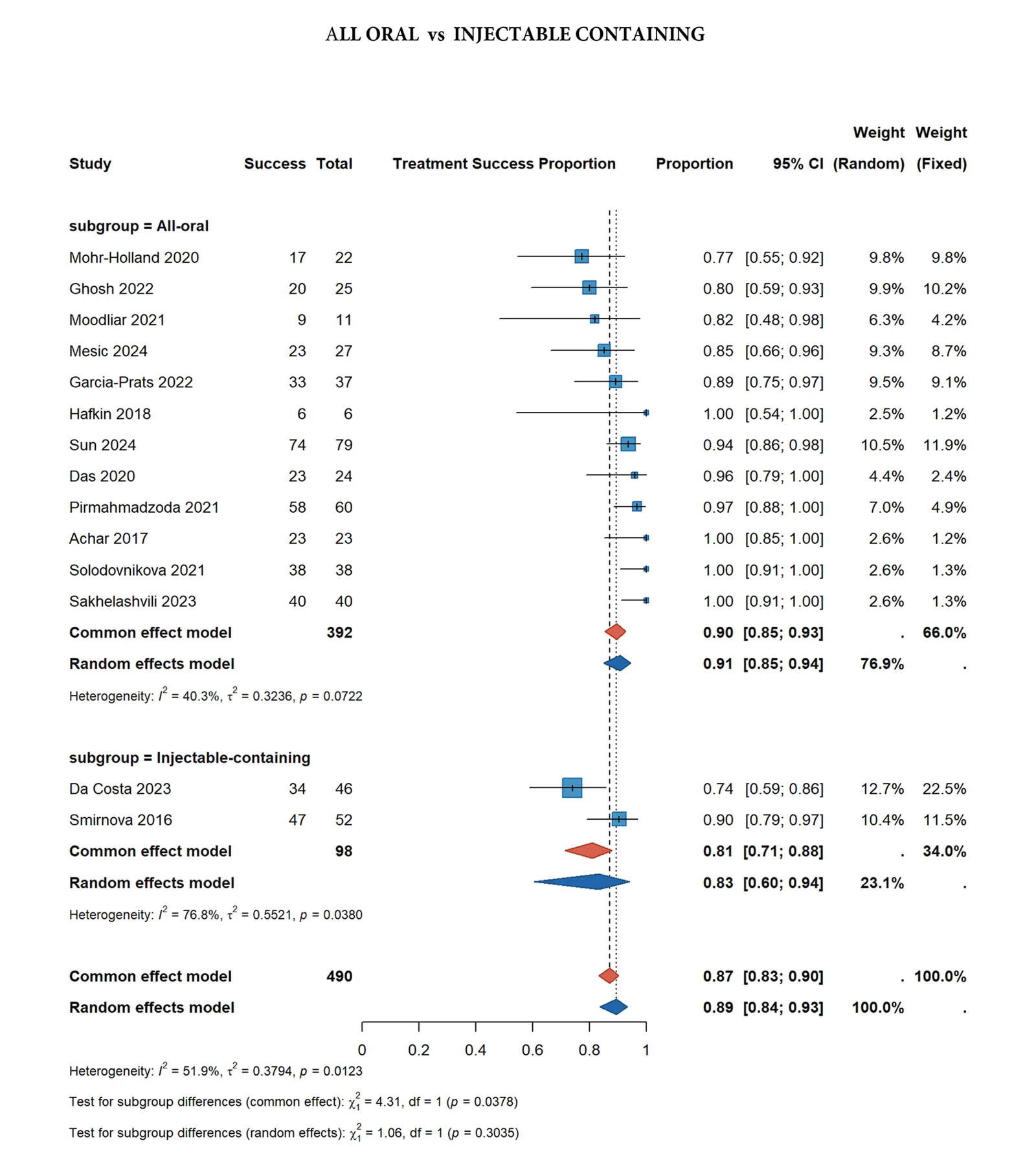
Forest plot illustrating treatment success by regimen type: all-oral versus injectable-containing regimens. CI indicates confidence interval.

### Sensitivity Analyses on Pooled Effectiveness

Sensitivity analyses confirmed robustness, with success proportions of 91% (95% CI: 84%–95%) when restricted to low-risk studies and 87% (95% CI: 79%–94%) when studies with 100% success were excluded (Figures, Supplemental Digital Content 8–10, https://links.lww.com/INF/G622). Reporting of extrapulmonary TB, as well as the proportion of XDR or pre-XDR cases and outcomes in children under 5 years, was sparse across studies, limiting subgroup analyses in these clinically important categories.

### Adverse Events

Only serious adverse events (CTCAE grade 3/4) were extracted, as these reflect severe or life-threatening toxicity most relevant to weighing the risks and benefits of MDR/XDR-TB treatment in children, and were reported in half of the included studies (7 of 14) (Fig. 3, Table, Supplemental Digital Content 11, https://links.lww.com/INF/G622). The pooled proportion of patients experiencing such events was 21% (95% CI: 13%–33%; *I*^2^=51.1%), with rates varying widely across studies. QT prolongation was the most frequently reported event, appearing in 3 studies and affecting up to 12.5% of patients, followed by anemia, eosinophilia, hepatic enzyme elevation and vomiting (Table, Supplemental Digital Content 11, https://links.lww.com/INF/G622). Where drug attribution was reported, anemia was linked to linezolid, cardiac events to bedaquiline, and hypomagnesemia and thrombocytopenia to delamanid, though most studies did not specify a causative drug. Notably, ototoxicity and nephrotoxicity, typically associated with injectable-containing regimens, were not reported among serious adverse events despite 2 studies including injectable-containing regimens. However, these toxicities were reported in the included studies but were generally of lower severity and did not meet the threshold for classification as serious adverse events. For example, in Da Costa et al,^36^ adverse reactions were observed in nearly half of participants, including mild renal toxicity and mild hearing loss, neither of which were classified as serious.^36^ Subgroup analyses comparing safety between bedaquiline-containing and delamanid-containing regimens, or between all-oral and injectable-containing regimens, were not possible due to sparse and inconsistent adverse event reporting; serious grade 3/4 events were documented in only 7 of 14 studies, and drug attribution was explicitly provided in only 3 of those (Fig. 3, Table, Supplemental Digital Content 11, https://links.lww.com/INF/G622).

**FIGURE 3.**
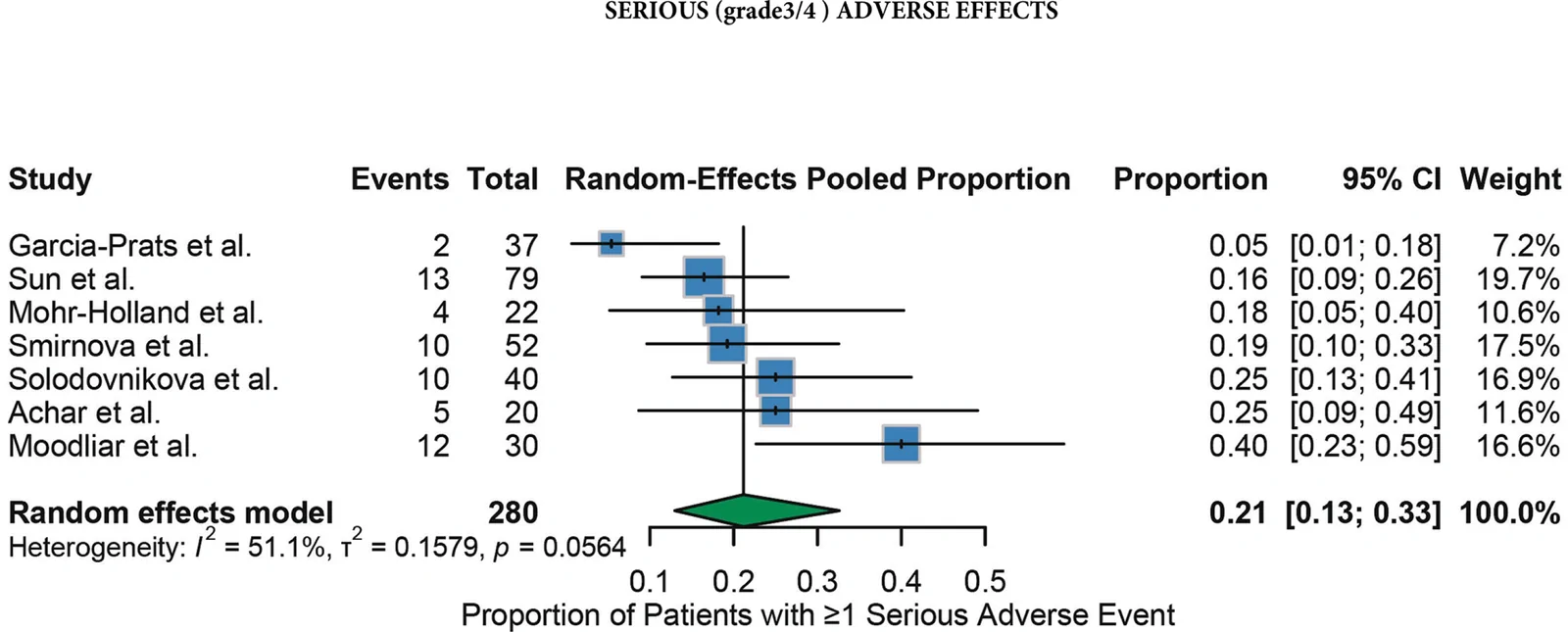
Forest plot illustrating the pooled proportion of patients experiencing serious adverse events (CTCAE grade 3/4) across included studies. CI indicates confidence interval; CTCAE, Common Terminology Criteria for Adverse Events.

### Bias and Certainty of Evidence

All 11 observational studies were rated as good quality with NOS scores ranging from 7 to 9, performing well in selection and outcome domains but generally weaker in comparability, with only Ghosh et al and Sun et al receiving full points for adjustment of confounding factors; all 3 clinical trials showed low risk of bias across key domains using the Cochrane Risk of Bias Tool (Table, Supplemental Digital Content 12, https://links.lww.com/INF/G622; Figure, Supplemental Digital Content 13, https://links.lww.com/INF/G622). Funnel plot in the overall pooled effectiveness and Egger’s regression test showed some potential small-study effects (*t* = 3.30, df = 12, *P* = 0.0063, Figures, Supplemental Digital Content 14–16, https://links.lww.com/INF/G622).

## DISCUSSION

### Summary of Key Findings

This contemporary systematic review and meta-analysis evaluated drug regimens for pediatric MDR- and XDR-TB across 14 studies involving 490 children from 13 countries. The pooled treatment success rate was 89% with moderate heterogeneity (*I*^2^ = 51.9%). Regimens using all oral combinations (91%), bedaquiline (92%) or delamanid (90%) showed slightly higher effectiveness than regimens with injectables (83%) or those without bedaquiline (84%) or delamanid (88%). All-oral short regimens of 12 months or less achieved slightly higher success (96%) than all-oral long regimens (84%) and injectable-containing long regimens (83%), with a significant difference between the 2 all-oral approaches. Treatment success was also slightly higher in low-income (93%) and non-high-burden (93%) settings than in upper- and middle-income (90%) and high-burden (90%) settings. Adverse events were reported in half of the studies. Among serious grade 3 or 4 events, QT prolongation and anemia were most common, while eosinophilia, vomiting, elevated hepatic enzymes, hypomagnesemia, thrombocytopenia and bronchial hyperreactivity were rare. The pooled proportion of serious grade 3 or 4 adverse events was 21%.

### Interpretation of Key Findings

The reviewed studies demonstrate consistently marginally high treatment success rates (overall 89%) for pediatric MDR/XDR-TB in the period from 2015 to date. Bedaquiline was initially approved and recommended by WHO for adult MDR-TB in 2013, with subsequent expansion of recommendations to adolescents and later to younger children in updated WHO guidelines (from 2019 onwards). Similarly, delamanid was approved for adults in 2014, with WHO recommendations for pediatric use introduced first for older children and adolescents and later expanded to younger age groups in subsequent guideline updates (from 2016 onwards).^16^ The proportions of success in our study, irrespective of regimen type, are slightly higher than or comparable to outcomes reported in previous systematic reviews based on injectable-containing regimens.^10,21^ In the first review, the pooled success rate for MDR-TB was 82%, while another review reported rates of 69% for pre-XDR-TB and 89% for XDR-TB.^10,21^ These findings suggest that all-oral regimens can achieve at least comparable—and often superior—treatment outcomes, representing a major advancement in pediatric MDR/XDR-TB care.

Studies reporting complete success (100%) included Solodovnikova et al (Belarus),^20^ Sakhelashvili et al (Ukraine),^27^ Achar et al (South Africa, Tajikistan, Uzbekistan),^30^ Hafkin et al (South Africa, India, Chile, USA),^32^ and Das et al (India, 96%).^29^ These studies predominantly utilized all-oral regimens including bedaquiline and/or delamanid combined with linezolid and fluoroquinolones.^20,27,29,30,32^ Bedaquiline disrupts bacterial adenosine triphosphate synthase, impairing energy metabolism,^37^ while delamanid inhibits mycolic acid synthesis, essential for bacterial cell wall integrity.^38^ Linezolid adds robust bactericidal effects but requires monitoring for hematologic side effects.^39^

Studies reporting relatively high success (80%–99%) included Sun et al (China, 94%),^22^ Mesic et al (Afghanistan, 85%),^19^ Garcia-Prats et al (Philippines, South Africa, 89%),^34^ Moodliar et al (Philippines, Russia, South Africa, 82%)^33^ and Ghosh et al (Russia, India, South Africa, 80%).^31^ These studies employed oral regimens typically containing bedaquiline or delamanid along with clofazimine, cycloserine, pyrazinamide and protionamide.^10,19,22,31,33,35^ Clofazimine provides anti-inflammatory and antimycobacterial benefits,^40^ whereas cycloserine interferes with bacterial cell wall synthesis.^41^ Fluoroquinolones (levofloxacin or moxifloxacin) contributed to regimen potency despite potential concerns about musculoskeletal effects in pediatric populations.^42^

Studies reporting moderate success (below 80%) included Mohr-Holland et al (South Africa, 77%)^28^ and Da Costa et al (Brazil, 74%).^36^ These studies faced challenges related to high HIV co-infection rates and undernutrition, complicating treatment outcomes.^36,43^ They frequently included injectable regimens featuring amikacin and capreomycin.^44,45^ The exception is Smirnova et al (Russia, 90%),^35^ which used injectables but reported comparably high success.

Subgroup analyses revealed slightly higher effectiveness in low-burden (93%) than high-burden (90%) settings. This indicates that oral regimens remain effective even under resource-constrained, high-burden conditions, supporting global applicability. Sensitivity analyses excluding studies with high risk of bias continued to show success above 80%, underscoring robustness. On the other hand, incomplete reporting of adjunctive antibiotics administered alongside bedaquiline and delamanid limited the precise analysis of regimen-specific effectiveness. Additionally, variations in TB monitoring protocols and culture-confirmation practices contributed to observed outcome variability. Nevertheless, the consistent favorable outcomes across studies (diverse settings and methodologies) underscore the effectiveness and generalizability of bedaquiline- and delamanid-based regimens. In contrast, individual participant data meta-analysis, as opposed to study-level aggregate data meta-analyses, has demonstrated the added value of patient-level adjustment and stratified analyses in clarifying heterogeneity of treatment results and identifying subgroup differences in response.^5,46^ Therefore, this study should be interpreted as a complementary descriptive synthesis, providing an overview of treatment effectiveness and reported adverse effects. The study-level meta-analysis approach in this study precluded adjustment for important prognostic factors such as the comorbidities. These factors are known to substantially influence treatment outcomes in pediatric MDR/XDR-TB and may vary considerably across settings and studies.^4,46^

Despite overall favorable outcomes, adverse effects remain an important consideration. Serious adverse events (CTCAE grade 3/4) were reported in half of the included studies, and where reported, they were generally infrequent, providing important insights into the safety profile of these regimens. Among the 7 studies that did report such events, QT prolongation and anemia were most frequently observed, both associated with drugs such as bedaquiline, linezolid and fluoroquinolones. Although serious, these events were generally manageable, as reflected by the high proportion of participants who successfully completed treatment. Other serious events, including hepatic enzyme elevation, hypomagnesemia and eosinophilia, were rare and highlight the importance of comprehensive laboratory and clinical monitoring. Attribution of adverse events to specific drugs was variably reported, reflecting differences in study design and available healthcare resources. Building on these findings, future research should prioritize standardized adverse event monitoring and harmonized pharmacovigilance frameworks to further strengthen the characterization of safety profiles of pediatric MDR/XDR-TB regimens.

### Strengths and Limitations

This review provides the most comprehensive synthesis to date of pediatric MDR/XDR-TB regimens involving newer drugs, drawing on studies from diverse geographic and economic contexts. The structured quality assessment enhances the external validity and credibility of the findings.

Pediatric TB research remains rare and chronically underfunded, underscoring the urgent need for greater global investment. This scarcity of evidence explains many of the limitations of the current literature. Most available studies were observational, with only a few randomized controlled trials, although their findings were broadly consistent, suggesting that the strong treatment effects observed may be robust. Sparse reporting of extrapulmonary TB and the proportion of specific resistance categories prevented subgroup analyses in these clinically important categories. Outcomes in children under 5 years of age, who face the highest risk of severe disease and rapid progression, were also rarely reported, limiting insights for this critical group. This review focused on serious CTCAE grade 3/4 adverse events, which are clinically significant and may require treatment modification or discontinuation, and may alter the course of treatment. This approach provides a clear and clinically meaningful assessment of safety by prioritizing events with the greatest impact on patient management. While milder grade 1/2 toxicities generally permit treatment continuation and were not systematically captured, the reported serious events offer valuable insight into clinically relevant safety outcomes. Serious grade 3/4 events were reported in 7 of 14 studies, with explicit drug attribution available in 3, enabling identification of key safety signals. Notably, injectable-related toxicities such as ototoxicity and nephrotoxicity were not reported among serious adverse events despite 2 studies including injectable-containing regimens; these toxicities were present and generally of lower severity, and did not meet the threshold for classification as serious. Documentation of regimen composition and duration, as well as adjustment for confounding variables, varied across studies and still allowed for meaningful interpretation of overall trends. Restriction to English-language publications may also have excluded relevant evidence. Furthermore, although studies including RR-TB patients were eligible and retained, disaggregated outcomes by resistance subtype (MDR-, XDR- or RR-TB) were not reported in most included studies, precluding a sensitivity analysis restricted to MDR/XDR-TB patients only.

In the meantime, as robust clinical trials in children with MDR/XDR-TB remain unavailable, this synthesis represents the best available evidence and can help guide clinical practice and policy.

## CONCLUSION

This meta-analysis shows that newer, all-oral bedaquiline- and delamanid-based regimens for pediatric MDR/XDR-TB have similar or slightly superior effectiveness compared with injectable-containing regimens, with available adverse event data suggesting that these regimens may be generally well tolerated. In pediatric MDR/XDR-TB, where studies are rare, these findings provide the best available evidence and a foundation for improved treatment guidelines and future research.

## ACKNOWLEDGMENTS

*We acknowledge Patience K. Phiri for helping with literature search.*

## Supplementary Material

## References

[1] Floyd K Glaziou P Zumla A. The global tuberculosis epidemic and progress in care, prevention, and research: an overview in year 3 of the end TB era. Lancet Respir Med. 2018;6:299–314. 10.1016/S2213-2600(18)30057-2 29595511

[2] Chakaya J Khan M Ntoumi F. Global tuberculosis report 2020—reflections on the global TB burden, treatment and prevention efforts. Int J Infect Dis. 2021;113:S7–S12. 10.1016/j.ijid.2021.02.107 PMC8433257 33716195

[3] Kirimuhuzya C. Multi-drug/extensively drug resistant tuberculosis (MDR/XDR-TB): renewed global battle against tuberculosis. In: Cardona PJ, ed. Understanding Tuberculosis—New Approaches to Fighting Against Drug Resistance. IntechOpen; 2012: 376.

[4] Jain A Mondal R. Extensively drug-resistant tuberculosis: current challenges and threats. FEMS Immunol Med Microbiol. 2008;53:145–150. 10.1111/j.1574-695X.2008.00400.x 18479439

[5] Mirzayev F Viney K Linh NN. World Health Organization recommendations on the treatment of drug-resistant tuberculosis, 2020 update. Eur Respir J. 2021;57:2003300. 10.1183/13993003.03300-2020 PMC8176349 33243847

[6] Monedero I Caminero JA. MDR-/XDR-TB management: what it was, current standards and what is ahead. Expert Rev Respir Med. 2009;3:133–145. 10.1586/ers.09.6 20477307

[7] Al-Worafi YM. Tuberculosis management in developing countries. In: Al-Worafi YM, ed. Handbook of Medical and Health Sciences in Developing Countries. Springer; 2024.

[8] Seaworth BJ Griffith DE. Therapy of Multidrug-Resistant and Extensively Drug-Resistant Tuberculosis. *Microbiol Spectr*. 2017;5:10.1128/microbiolspec.tnmi7-0042-2017. 10.1128/microbiolspec.tnmi7-0042-2017 PMC11687483 28361737

[9] World Health Organization. WHO Global Lists of High Burden Countries for Tuberculosis (TB), TB/HIV and Multidrug/Rifampicin-Resistant TB (MDR/RR-TB), 2021–2025: Background Document. World Health Organization; 2021.

[10] Garcia-Prats AJ Garcia-Cremades M Cox V; endTB Study Group. Characteristics of children and adolescents with multidrug-resistant and rifampicin-resistant tuberculosis and their association with treatment outcomes: a systematic review and individual participant data meta-analysis. Lancet Child Adolesc Health. 2025;9:100–111. 10.1016/S2352-4642(24)00330-4 PMC13289721 39855750

[11] Chowdhury K Ahmad R Sinha S. Multidrug-resistant TB (MDR-TB) and extensively drug-resistant TB (XDR-TB) among children: where we stand now. Cureus. 2023;15 :e35154. 10.7759/cureus.35154 PMC9938784 36819973

[12] Moore DP. Childhood tuberculosis guidelines of the Southern African Society for Paediatric Infectious Diseases: guidelines. South Afr J Epidemiol Infect. 2009;24:57–68.

[13] Golla V Snow K Mandalakas AM. The impact of drug resistance on the risk of tuberculosis infection and disease in child household contacts: a cross sectional study. BMC Infect Dis. 2017;17:1–10. 10.1186/s12879-017-2668-2 PMC5576070 28851285

[14] Song WM Li YF Liu YX. Drug-resistant tuberculosis among children: a systematic review and meta-analysis. Front Public Health. 2021;9:721817. 10.3389/fpubh.2021.721817 PMC8416474 34490197

[15] World Health Organization. WHO Operational Handbook on Tuberculosis. Module 4: Treatment-Tuberculosis Care and Support. World Health Organization; 2022.

[16] Dheda K Cox H Esmail A. Recent controversies about MDR and XDR‐TB: Global implementation of the WHO shorter MDR‐TB regimen and bedaquiline for all with MDR‐TB? Respirology. 2018;23:36–45. 10.1111/resp.13143 28850767

[17] Pontali E Raviglione MC Migliori GB; and the writing group members of the Global TB Network Clinical Trials Committee. Regimens to treat multidrug-resistant tuberculosis: past, present and future perspectives. Eur Respir Rev. 2019;28:190035. 10.1183/16000617.0035-2019 PMC9489134 31142549

[18] Pecora F Dal Canto G Veronese P. Treatment of multidrug-resistant and extensively drug-resistant tuberculosis in children: the role of bedaquiline and delamanid. Microorganisms. 2021;9:1074. 10.3390/microorganisms9051074 PMC8156326 34067732

[19] Mesic A Decuyper I Ishaq S. Short oral treatment regimens for rifampicin-resistant tuberculosis are safe and effective for young children: results from a field-based non-randomized clinical trial, Kandahar Afghanistan. Eur Respir J. 2024;63:2400436. 10.1183/13993003.00436-2024 PMC11137330 38782466

[20] Solodovnikova V Kumar AM Hurevich H. Effectiveness and safety of delamanid-or bedaquiline-containing regimens among children and adolescents with multidrug resistant or extensively drug resistant tuberculosis: a nationwide study from Belarus, 2015-19. Monaldi Arch Chest Dis. 2021;91:e1646. 10.4081/monaldi.2021.1646 33470080

[21] Patra J Irving H Maini P. Treatment outcomes among children and adolescents with extensively drug–resistant (XDR) and pre–XDR tuberculosis: systematic review and meta–analysis. PLOS Global Public Health. 2025;5:e0003754. 10.1371/journal.pgph.0003754 PMC11778756 39879191

[22] Sun W-W Yang M Chen X-H. Efficacy and safety of the all-oral bedaquiline-containing regimen as treatment for pediatric multidrug/rifampicin-resistant tuberculosis: a multicenter, retrospective, cohort study. Expert Rev Anti Infect Ther. 2024;22:219–227. 10.1080/14787210.2023.2285917 37982155

[23] World Health Organization. WHO Consolidated Guidelines on Tuberculosis. Module 4: Treatment-Drug-Resistant Tuberculosis Treatment, 2022 Update. World Health Organization; 2022. 36630546

[24] Colevas AD Setser A. The NCI common terminology criteria for adverse events (CTCAE) v 3.0 is the new standard for oncology clinical trials. J Clin Oncol. 2004;22(14_suppl):6098.

[25] Wells GA Shea B O’Connell D. The Newcastle-Ottawa Scale (NOS) for assessing the quality of nonrandomised studies in meta-analyses. 2000.

[26] Corbett MS Higgins JP Woolacott NF. Assessing baseline imbalance in randomised trials: implications for the Cochrane Risk of Bias tool. Res Synth Methods. 2014;5:79–85. 10.1002/jrsm.1090 26054027

[27] Sakhelashvili-Bil OI Platonova IL. Evaluation of the effectiveness of immunomodulator BI-V in the complex therapy of children and adolescents with multiple drug-resistant pulmonary tuberculosis. Infus Chemother. 2023;6:31–35.

[28] Mohr-Holland E Reuter A Furin J. Injectable-free regimens containing bedaquiline, delamanid, or both for adolescents with rifampicin-resistant tuberculosis in Khayelitsha, South Africa. EClinicalMedicine. 2020;20:100290. 10.1016/j.eclinm.2020.100290 PMC7057194 32154506

[29] Das M Mamnoon F Mansoor H. New TB drugs for the treatment of children and adolescents with rifampicin-resistant TB in Mumbai, India. Int J Tuberc Lung Dis. 2020;24:1265–1271. 10.5588/ijtld.20.0165 33317670

[30] Achar J Hewison C Cavalheiro AP. Off-label use of bedaquiline in children and adolescents with multidrug-resistant tuberculosis. Emerg Infect Dis. 2017;23:1711–1713. 10.3201/eid2310.170303 PMC5621552 28758889

[31] Ghosh S Breitscheidel L Lazarevic N. Compassionate use of delamanid in adults and children for drug-resistant tuberculosis: 5-year update. Eur Respir J. 2021;57:2002483. 10.1183/13993003.02483-2020 33243846

[32] Hafkin J Hittel N Martin A. Compassionate use of delamanid in combination with bedaquiline for the treatment of MDR-TB. Eur Respir J. 2018;53:1801154. 10.1183/13993003.01154-2018 30361253

[33] Moodliar R Aksenova V Frias MVG. Bedaquiline for multidrug-resistant TB in paediatric patients. Int J Tuberc Lung Dis. 2021;25:716–724. 10.5588/ijtld.21.0022 PMC8412106 34802493

[34] Garcia-Prats AJ Frias M van der Laan L. Delamanid added to an optimized background regimen in children with multidrug-resistant tuberculosis: results of a phase I/II clinical trial. Antimicrob Agents Chemother. 2022;66:e0214421. 10.1128/aac.02144-21 PMC9112969 35404075

[35] Smirnova PA Turkova A Nikishova EI. Multidrug-resistant tuberculosis in children in northwest Russia: an observational cohort study. Eur Respir J. 2016;48:1496–1499. 10.1183/13993003.00354-2016 27587542

[36] Bruzadelli Paulino da Costa F Zamboni Berra T Garcia de Almeida Ballestero J. Treatment of drug-resistant tuberculosis in children and young adolescents in Brazil. J Clin Tuberc Other Mycobact Dis. 2023;33:100388. 10.1016/j.jctube.2023.100388 PMC10425933 37588725

[37] Nguyen TVA Anthony RM Bañuls A-L. Bedaquiline resistance: its emergence, mechanism, and prevention. Clin Infect Dis. 2018;66:1625–1630. 10.1093/cid/cix992 29126225

[38] Liu Y Matsumoto M Ishida H. Delamanid: from discovery to its use for pulmonary multidrug-resistant tuberculosis (MDR-TB). Tuberculosis (Edinb). 2018;111:20–30. 10.1016/j.tube.2018.04.008 30029909

[39] Vinh DC Rubinstein E. Linezolid: a review of safety and tolerability. J Infect. 2009;59:S59–S74. 10.1016/S0163-4453(09)60009-8 19766891

[40] Cholo MC Steel HC Fourie PB. Clofazimine: current status and future prospects. J Antimicrob Chemother. 2012;67:290–298. 10.1093/jac/dkr444 22020137

[41] Epstein IG Nair K Boyd LJ. The treatment of human pulmonary tuberculosis with cycloserine:* progress report. Dis Chest. 1956;29:241–257. 10.1378/chest.29.3.241 13294011

[42] Drlica K Malik M. Fluoroquinolones: action and resistance. Curr Top Med Chem. 2003;3:249–282. 10.2174/1568026033452537 12570763

[43] Mohr-Holland E Douglas-Jones B Apolisi I. Tuberculosis preventive therapy for children and adolescents: an emergency response to the COVID-19 pandemic. Lancet Child Adolesc Health. 2021;5:159–161. 10.1016/S2352-4642(21)00003-1 PMC9765870 33609490

[44] Ramirez MS Tolmasky ME. Amikacin: uses, resistance, and prospects for inhibition. Molecules. 2017;22:2267. 10.3390/molecules22122267 PMC5889950 29257114

[45] Stanley RE Blaha G Grodzicki RL. The structures of the anti-tuberculosis antibiotics viomycin and capreomycin bound to the 70S ribosome. Nat Struct Mol Biol. 2010;17:289–293. 10.1038/nsmb.1755 PMC2917106 20154709

[46] Rai E Naik V Williams A. Individual participant data (IPD) meta-analysis: an introduction–narrative review. Indian J Anaesth. 2025;69:153–160. 10.4103/ija.ija_1187_24 PMC11878366 40046704

