# Supplementary material for: Effectiveness and Safety of Contemporary Drug Regimens for Multidrug- and Extensively Drug-resistant Tuberculosis in Children and Adolescents: A Global Systematic Review and Meta-analysis
Source: Pediatr Infect Dis J. 2026 May 21;45(11):1003–12. doi: 10.1097/INF.0000000000005269 (PMC13649270; doi:10.1097/INF.0000000000005269)

Supplemental Digital Content 1. PRISMA flow chart depicting the study selection process.

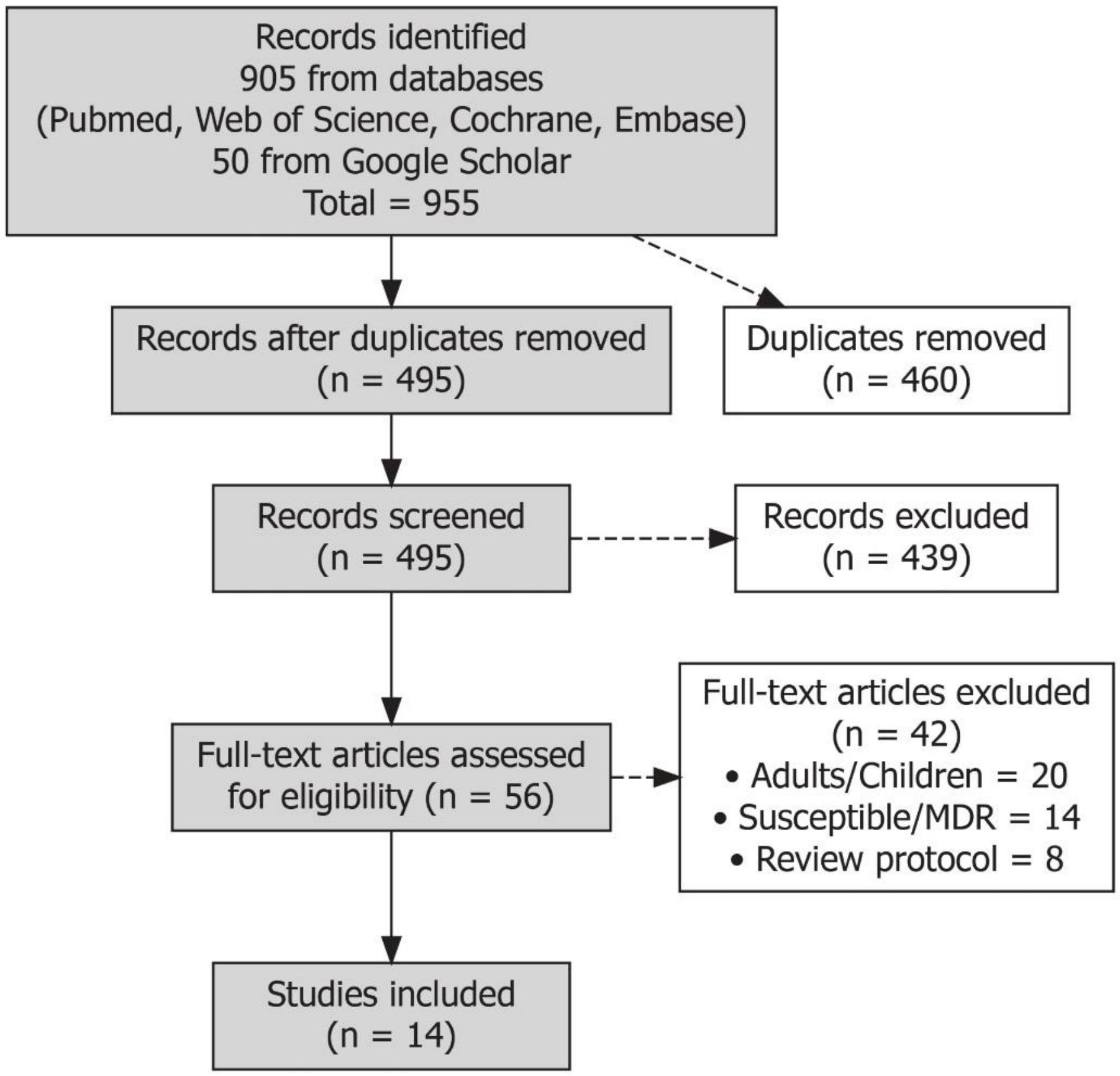

Supplement: Supplementary file 1 [file inf-45-1003-s001.pdf]
