# Supplementary material for: Effectiveness and Safety of Contemporary Drug Regimens for Multidrug- and Extensively Drug-resistant Tuberculosis in Children and Adolescents: A Global Systematic Review and Meta-analysis
Source: Pediatr Infect Dis J. 2026 May 21;45(11):1003–12. doi: 10.1097/INF.0000000000005269 (PMC13649270; doi:10.1097/INF.0000000000005269)
Supplement: Supplementary file 2 [file inf-45-1003-s002.pdf]

SUPPLEMENTAL DIGITAL CONTENT 2. Characteristics of studies included in analysis

No.	Author, Year, Study Country	Age Range (years)	< 5 years old %	Female %	TB dx method	Extra Pulmonary TB	XDR %	Co-morbidity	Comparison group	Definition of treatment success
**1**	Solodovnikova et al. 2021, Belarus	10 to 17	None	55%	Sputum microscopy, Xpert MTB/RIF assay, Line Probe Assay, Chest radiography	Not reported	50%	Anaemia, Epilepsy, HIV, Myopia	No control group	Cured and treatment completed. Cured patients had two consecutive culture-negative results at least 30 days apart.
**2**	Sakhelashvili et al 2023, Ukraine	0 to 17	Not given	Not Given	Microbiological, Sputum smear, culture on nutrient media, and radiographic evaluation.	All Pulmonary	Not given	Not mentioned	27 children receiving antimycobacterial therapy (AMBT) without Bdq and Dlm.	Cured or completed treatment
**3**	Mohr-Holland et al 2020, South Africa	10 to 19	None	41%	Sputum Culture	9.1%	None	6 HIV positive patients	No control group	Cured or completed treatment
**4**	Das et al 2020, India	3 to 18	Not given	63%	Sputum culture	Not given	Not given	No HIV co-morbidity	No control group	Cured or Completed treatment
**5**	Achar et al 2017, South Africa, Tajikistan, and Uzbekistan	10 to 17	None	56%	Sputum culture confirmation	Not given	67%	Intrathoracic lymph node TB	No explicit control group	Culture conversion at end of treatment

No.	Author, Year, Study Country	Age Range (years)	< 5 years old %	Female %	TB dx method	Extra Pulmonary TB	XDR %	Co-morbidity	Comparison group	Definition of treatment success
**6**	Pirmahmadzoda et al 2021, Tajikistan	0 to 17	Not given	39%	BacteriologicalSputum smear Xpert MTB/RIF	Not given	Not given	1 person living with HIV	No direct control group, but different regimens were compared	Cured (Culture conversion) or treatment completed
**7**	Ghosh et al 2022, Russia, India, South Africa	6 to 17	None	Not Given	Sputum culture	Not given	Not given	people living with HIV (PLWH), HCV, HBV, Diabetes mellitus	No control group	Culture conversion at 24 weeks
**8**	Hafkin et al 2018, South Africa, India, Chile, USA	8 to 17	Not given	33%	Not explicitly mentioned for the paediatric group.	Not given	Not given	Not explicitly mentioned for the paediatric group.	No control group	Culture-negative at the end of delamanid treatment
**9**	Moodliar et al 2021, Philippines, Russia, South Africa	5 to 17	Not given	70%	MGIT culture	Not given	Not given	Not mentioned	No control group	Culture conversion at end of treatment
**10**	Garcia-Prats et al 2022, Philippines and South Africa	0 to 17	64%	52%	Clinical diagnosis	Not given	Not given	Not mentioned	No control group	Cured: Had at least three consecutive negative cultures taken at least 30 days apart in the final 12 months of treatment.

No.	Author, Year, Study Country	Age Range (years)	< 5 years old %	Female %	TB dx method	Extra Pulmonary TB	XDR %	Co-morbidity	Comparison group	Definition of treatment success
**11**	Smirnova et al 2016, Russia	0 to <15<br>15 to 18	Not given	60%	Clinical diagnosis	58%	None	No people living with HIV or other co-morbidities	No control group	Cured or treatment completed
**12**	Da Costa et al. 2023, Brazil	0 to 14	15%	35%	Bacilloscopy, Culture, Xpert MTB/RIF (Rapid Molecular Test), Chest X-ray	17.4%	2.2%	people living with HIV (PLWH), Underweight children	No distinct control group	Cured or completed treatment
**13**	Sun et al. 2024, China	10 to 17		40% in the BDQ group 48% in the control group	Sputum or bronchoalveolar lavage fluid (BALF) culture			1 Diabetes patient	42 children receiving an injection-containing regimen instead of an all-oral regimen	At least three consecutive negative TB cultures (spaced 30 days apart).
**14**	Mesic et al. 2024, Afghanistan	0 to 14	Not given	45%	Microbiological confirmation radiographic signs	11%	(29.6%) pre-XDR-TB	No mention	No control group, but compared to prior longer, injectable-based regimens.	Completed treatment or were cured

Definition of cured or completed treatment is based on The World Health Organization (WHO) definitions of successful TB treatment outcomes. For cured patients; at least three consecutive negative cultures, taken at least 30 days apart in the final 12 months of treatment. Completed treatment means the patient did not have lab confirmation but they must finish the full treatment course without evidence of failure. Bdq = bedaquiline; Dlm = delamanid; Lzd = linezolid; Cfz = clofazimine; FQ = fluoroquinolone; Mfx =

moxifloxacin; Lfx = levofloxacin; Imi = imipenem; Cs = cycloserine; Pto = protionamide; PAS = para-aminosalicylic acid; Cm = capreomycin; Am = amikacin; EMB = ethambutol; PZA or Z = pyrazinamide; Trd = terizidone.

SUPPLEMENTAL DIGITAL CONTENT 3. Bedaquiline (BDQ) vs No Bedaquiline

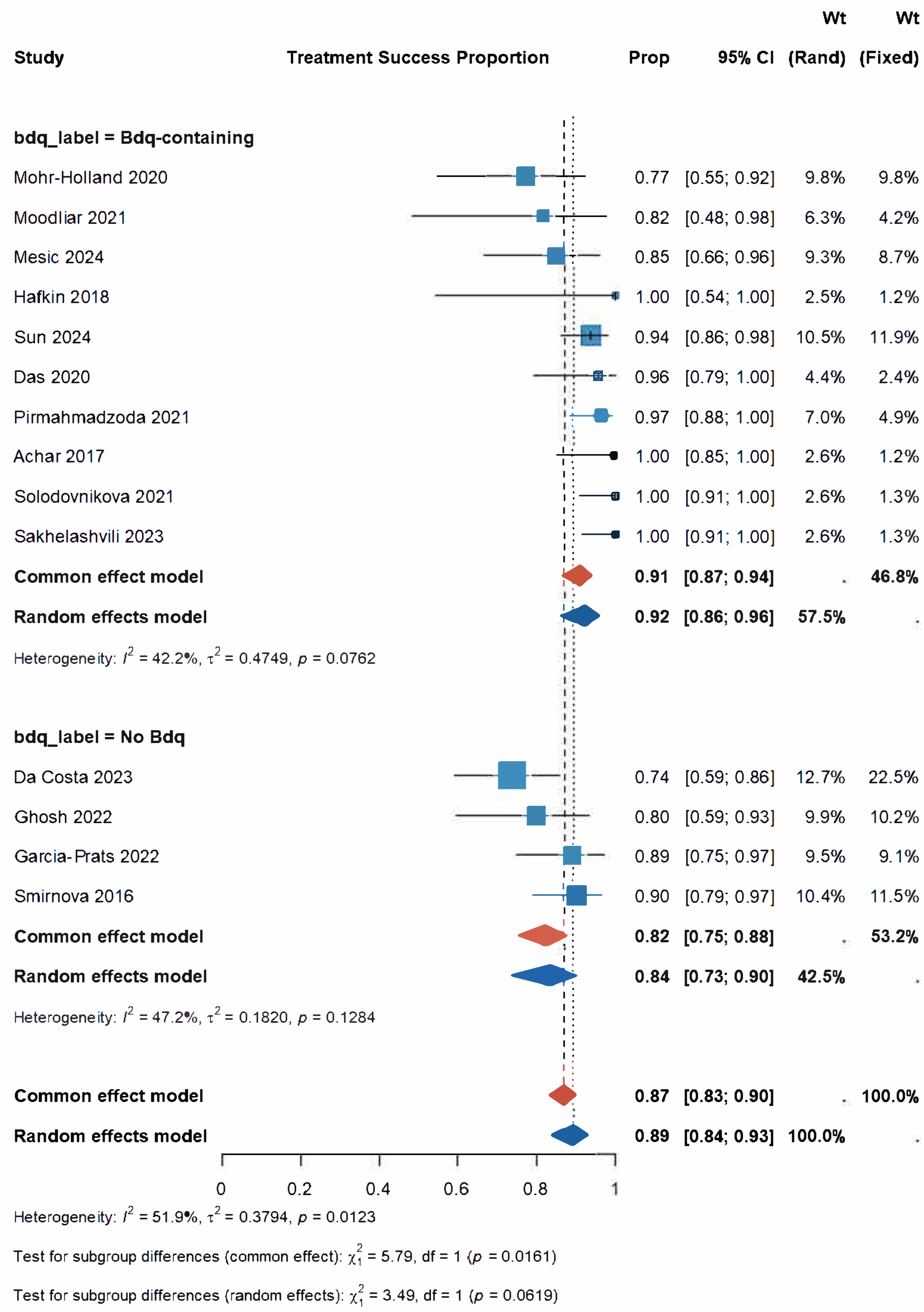

SUPPLEMENTAL DIGITAL CONTENT 4. Delamanid (DLM) vs No Delamanid

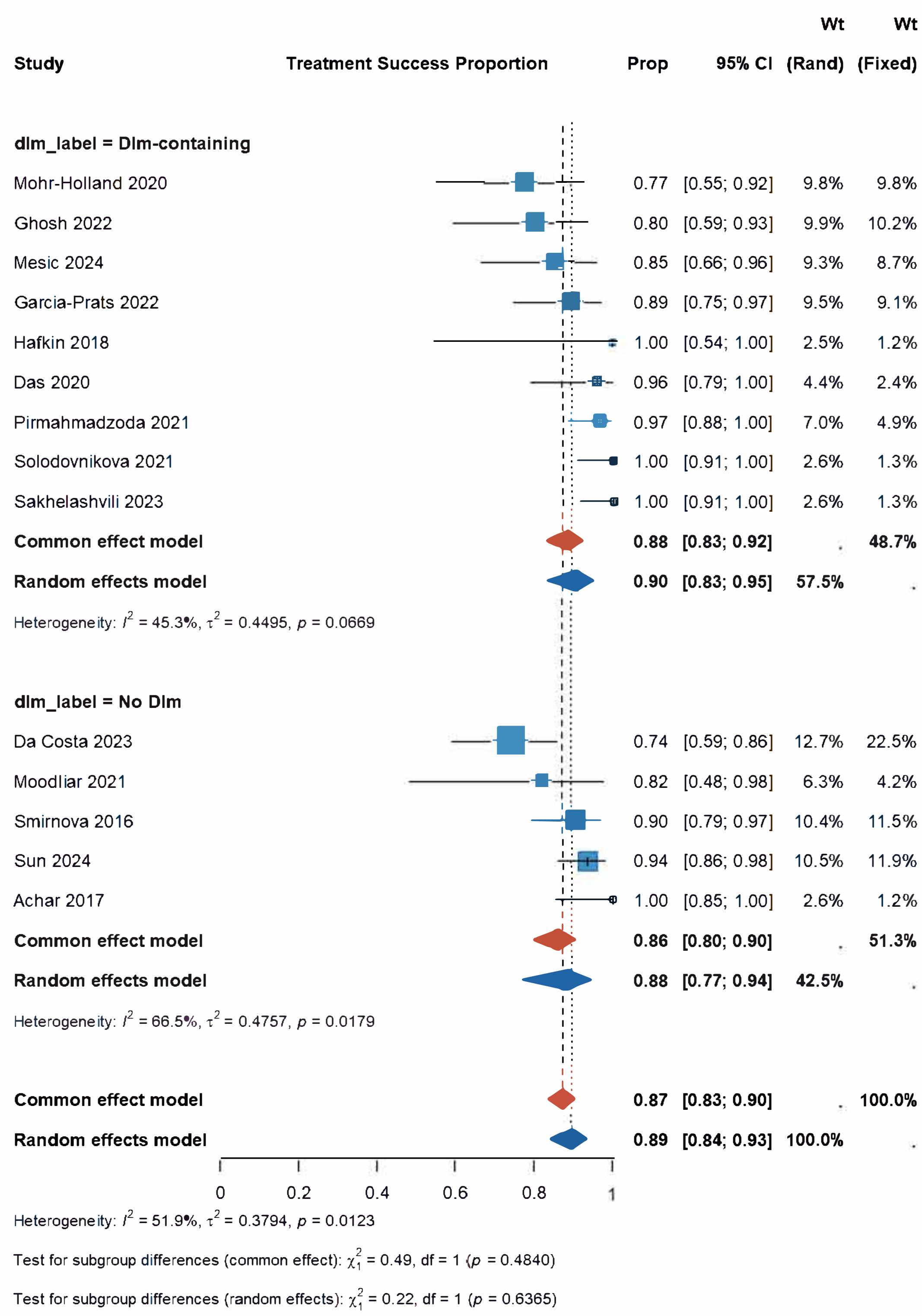

SUPPLEMENTAL DIGITAL CONTENT 5. Short Duration vs Long Duration

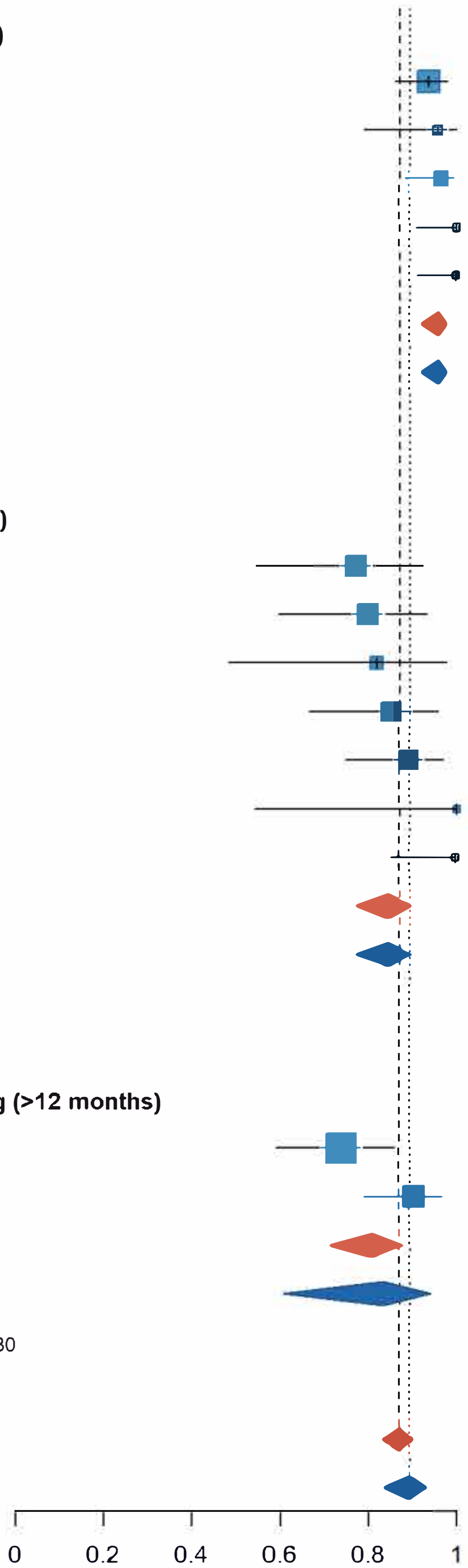

Study	Success	Total	Proportion	95% CI	Weight (Random)	Weight (Fixed)
**subgroup = All-oral, Long (>12 months)**						
Sun 2024	74	79	0.94	[0.86; 0.98]	10.5%	11.9%
Das 2020	23	24	0.96	[0.79; 1.00]	4.4%	2.4%
Pirmahmadzoda 2021	58	60	0.97	[0.88; 1.00]	7.0%	4.9%
Solodovnikova 2021	38	38	1.00	[0.91; 1.00]	2.6%	1.3%
Sakhelashvili 2023	40	40	1.00	[0.91; 1.00]	2.6%	1.3%
**Common effect model**		**241**	**0.96**	**[0.92; 0.98]**	.	**21.7%**
**Random effects model**			**0.96**	**[0.92; 0.98]**	**27.1%**	.
Heterogeneity: $I^2 = 0\%$, $\tau^2 = 0$, $p = 0.6448$						
**subgroup = All-oral, Short (≤12 months)**						
Mohr-Holland 2020	17	22	0.77	[0.55; 0.92]	9.8%	9.8%
Ghosh 2022	20	25	0.80	[0.59; 0.93]	9.9%	10.2%
Moodliar 2021	9	11	0.82	[0.48; 0.98]	6.3%	4.2%
Mesic 2024	23	27	0.85	[0.66; 0.96]	9.3%	8.7%
Garcia-Prats 2022	33	37	0.89	[0.75; 0.97]	9.5%	9.1%
Hafkin 2018	6	6	1.00	[0.54; 1.00]	2.5%	1.2%
Achar 2017	23	23	1.00	[0.85; 1.00]	2.6%	1.2%
**Common effect model**		**151**	**0.84**	**[0.77; 0.90]**	.	**44.3%**
**Random effects model**			**0.84**	**[0.77; 0.90]**	**49.8%**	.
Heterogeneity: $I^2 = 0\%$, $\tau^2 = 0$, $p = 0.6032$						
**subgroup = Injectable-containing, Long (>12 months)**						
Da Costa 2023	34	46	0.74	[0.59; 0.86]	12.7%	22.5%
Smirnova 2016	47	52	0.90	[0.79; 0.97]	10.4%	11.5%
**Common effect model**		**98**	**0.81**	**[0.71; 0.88]**	.	**34.0%**
**Random effects model**			**0.83**	**[0.60; 0.94]**	**23.1%**	.
Heterogeneity: $I^2 = 76.8\%$, $\tau^2 = 0.5521$, $p = 0.0380$						
**Common effect model**		**490**	**0.87**	**[0.83; 0.90]**	.	**100.0%**
**Random effects model**			**0.89**	**[0.84; 0.93]**	**100.0%**	.

Heterogeneity: $I^2 = 51.9\%$, $\tau^2 = 0.3794$, $p = 0.0123$

Test for subgroup differences (common effect): $\chi^2_2 = 15.69$, df = 2 ($p = 0.0004$)

Test for subgroup differences (random effects): $\chi^2_2 = 12.14$, df = 2 ($p = 0.0023$)

SUPPLEMENTAL DIGITAL CONTENT 6. Differences by income group

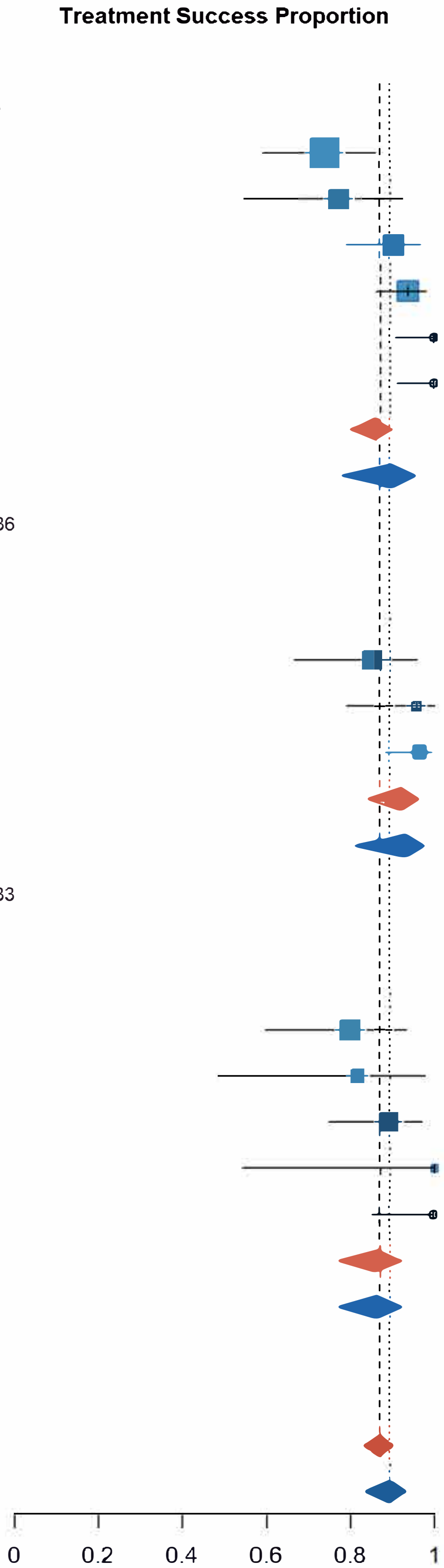

Study	Success	Total	Proportion	95% CI	Weight (Random)	Weight (Fixed)
**subgroup = Upper-middle/High income**						
Da Costa 2023	34	46	0.74	[0.59; 0.86]	12.7%	22.5%
Mohr-Holland 2020	17	22	0.77	[0.55; 0.92]	9.8%	9.8%
Smirnova 2016	47	52	0.90	[0.79; 0.97]	10.4%	11.5%
Sun 2024	74	79	0.94	[0.86; 0.98]	10.5%	11.9%
Solodovnikova 2021	38	38	1.00	[0.91; 1.00]	2.6%	1.3%
Sakhelashvili 2023	40	40	1.00	[0.91; 1.00]	2.6%	1.3%
**Common effect model**		**277**	**0.86**	**[0.80; 0.90]**	.	**58.2%**
**Random effects model**			**0.90**	**[0.78; 0.96]**	**48.6%**	.
Heterogeneity: $I^2 = 71.5\%$, $\tau^2 = 0.8047$, $p = 0.0036$						
**subgroup = Low/Lower-middle income**						
Mesic 2024	23	27	0.85	[0.66; 0.96]	9.3%	8.7%
Das 2020	23	24	0.96	[0.79; 1.00]	4.4%	2.4%
Pirmahmadzoda 2021	58	60	0.97	[0.88; 1.00]	7.0%	4.9%
**Common effect model**		**111**	**0.92**	**[0.84; 0.96]**	.	**16.0%**
**Random effects model**			**0.93**	**[0.81; 0.98]**	**20.6%**	.
Heterogeneity: $I^2 = 46.7\%$, $\tau^2 = 0.4934$, $p = 0.1533$						
**subgroup = Mixed/Multi-country**						
Ghosh 2022	20	25	0.80	[0.59; 0.93]	9.9%	10.2%
Moodliar 2021	9	11	0.82	[0.48; 0.98]	6.3%	4.2%
Garcia-Prats 2022	33	37	0.89	[0.75; 0.97]	9.5%	9.1%
Hafkin 2018	6	6	1.00	[0.54; 1.00]	2.5%	1.2%
Achar 2017	23	23	1.00	[0.85; 1.00]	2.6%	1.2%
**Common effect model**		**102**	**0.86**	**[0.77; 0.92]**	.	**25.8%**
**Random effects model**			**0.86**	**[0.77; 0.92]**	**30.7%**	.
Heterogeneity: $I^2 = 0\%$, $\tau^2 = 0$, $p = 0.4794$						
**Common effect model**		**490**	**0.87**	**[0.83; 0.90]**	.	**100.0%**
**Random effects model**			**0.89**	**[0.84; 0.93]**	**100.0%**	.

Heterogeneity: $I^2 = 51.9\%$, $\tau^2 = 0.3794$, $p = 0.0123$

Test for subgroup differences (common effect): $\chi^2_2 = 2.28$, df = 2 ($p = 0.3205$)

Test for subgroup differences (random effects): $\chi^2_2 = 1.46$, df = 2 ($p = 0.4809$)

SUPPLEMENTAL DIGITAL CONTENT 7. Differences by TB Burden in setting

Study	Success	Total	Proportion	95% CI	Weight (Random)	Weight (Fixed)
**subgroup = Non-high TB burden**						
Da Costa 2023	34	46	0.74	[0.59; 0.86]	12.7%	22.5%
Smirnova 2016	47	52	0.90	[0.79; 0.97]	10.4%	11.5%
Solodovnikova 2021	38	38	1.00	[0.91; 1.00]	2.6%	1.3%
Sakhelashvili 2023	40	40	1.00	[0.91; 1.00]	2.6%	1.3%
**Common effect model**		**176**	**0.84**	**[0.76; 0.90]**	**.**	**36.5%**
**Random effects model**			**0.93**	**[0.73; 0.98]**	**28.3%**	**.**
Heterogeneity: I^2 = 75.3%, τ^2 = 1.7639, p = 0.0068						
**subgroup = High TB burden**						
Mohr-Holland 2020	17	22	0.77	[0.55; 0.92]	9.8%	9.8%
Mesic 2024	23	27	0.85	[0.66; 0.96]	9.3%	8.7%
Garcia-Prats 2022	33	37	0.89	[0.75; 0.97]	9.5%	9.1%
Sun 2024	74	79	0.94	[0.86; 0.98]	10.5%	11.9%
Das 2020	23	24	0.96	[0.79; 1.00]	4.4%	2.4%
Pirmahmadzoda 2021	58	60	0.97	[0.88; 1.00]	7.0%	4.9%
**Common effect model**		**249**	**0.90**	**[0.85; 0.93]**	**.**	**46.8%**
**Random effects model**			**0.90**	**[0.83; 0.95]**	**50.4%**	**.**
Heterogeneity: I^2 = 44.6%, τ^2 = 0.2742, p = 0.1082						
**subgroup = Mixed/Ambiguous**						
Ghosh 2022	20	25	0.80	[0.59; 0.93]	9.9%	10.2%
Moodliar 2021	9	11	0.82	[0.48; 0.98]	6.3%	4.2%
Hafkin 2018	6	6	1.00	[0.54; 1.00]	2.5%	1.2%
Achar 2017	23	23	1.00	[0.85; 1.00]	2.6%	1.2%
**Common effect model**		**65**	**0.84**	**[0.71; 0.92]**	**.**	**16.7%**
**Random effects model**			**0.84**	**[0.71; 0.92]**	**21.3%**	**.**
Heterogeneity: I^2 = 2.1%, τ^2 = < 0.0001, p = 0.3817						
**Common effect model**		**490**	**0.87**	**[0.83; 0.90]**	**.**	**100.0%**
**Random effects model**			**0.89**	**[0.84; 0.93]**	**100.0%**	**.**

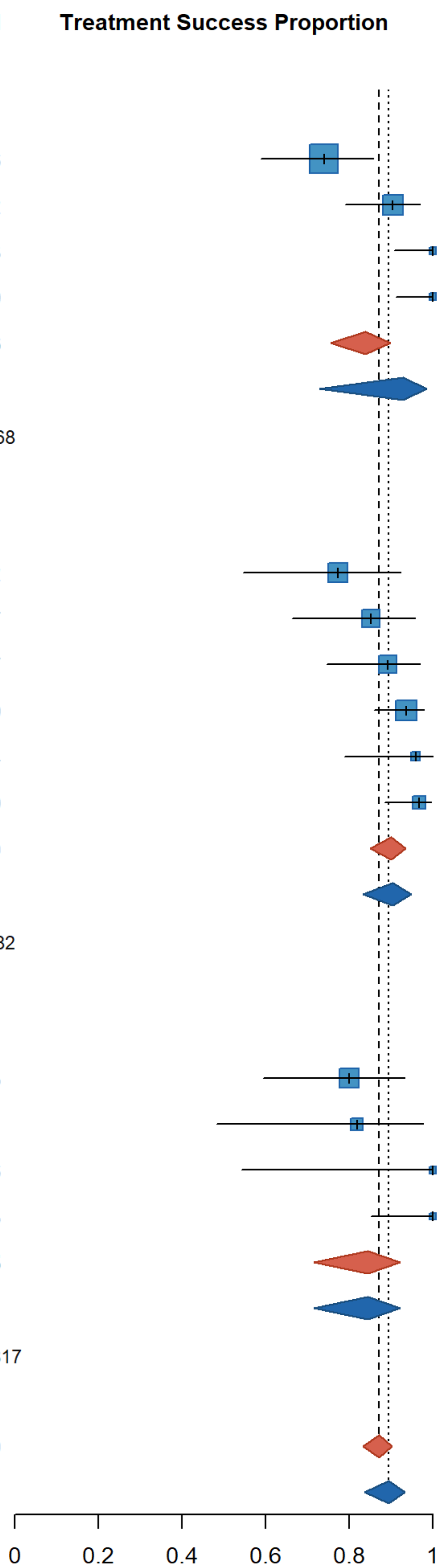

Heterogeneity: I^2 = 51.9%, τ^2 = 0.3794, p = 0.0123

Test for subgroup differences (common effect): χ^2_2 = 2.80, df = 2 (p = 0.2471)

Test for subgroup differences (random effects): χ^2_2 = 1.64, df = 2 (p = 0.4406)

SUPPLEMENTAL DIGITAL CONTENT 8. Sensitivity analysis (100% success excluded)

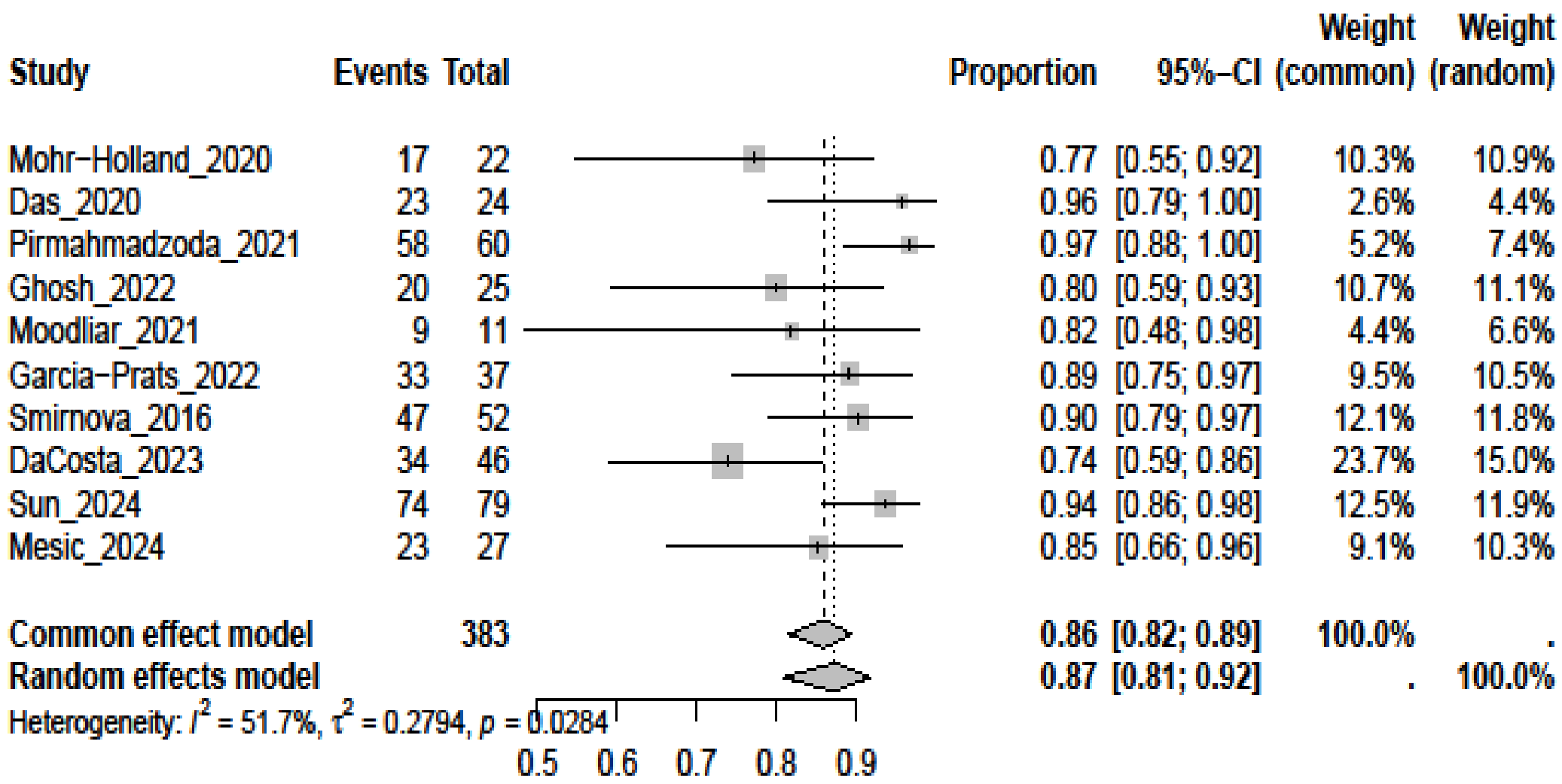

SUPPLEMENTAL DIGITAL CONTENT 9. Sensitivity analysis (inclusion of low risk of bias only)

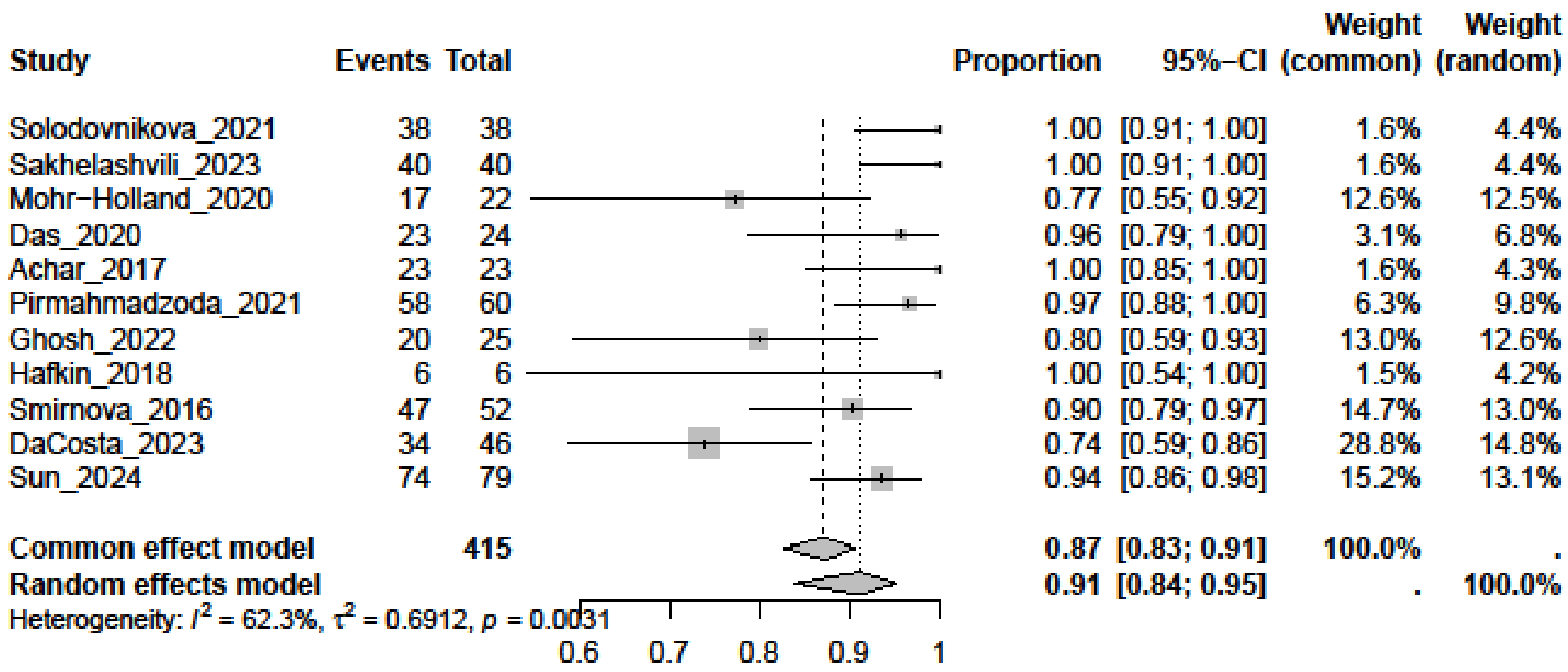

SUPPLEMENTAL DIGITAL CONTENT 10. Sensitivity analysis (inclusion of low risk of bias only and exclusion of 100% success)

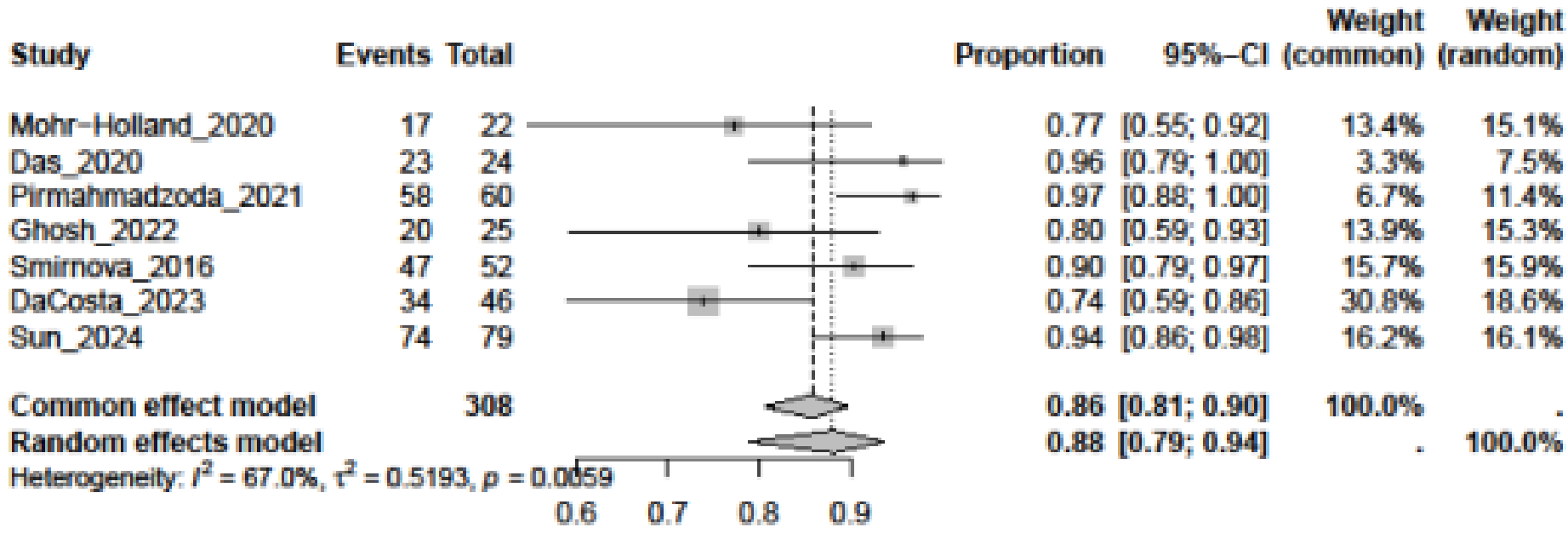

SUPPLEMENTAL DIGITAL CONTENT 11. Summary of Adverse Events Classified as Serious of CTCAE Grade 3-4

No.	Author	Drug regimen	Total (n/N)	Anemia	Eosinophilia	QT elongation	Vomiting	Hepatic enzyme ↑	Hypo-magnesemia	Thrombo-cytopenia	Bronco Hyper reactivity	Attribute
1	Solodovnikova et al.	FQ, Bdq, Dlm, Lzd	10/40 (25%)	–	2/40 (5%)	5/40 (12.5%)	1/40 (2.5%)	1/40 (2.5%)	–	–	–	Not specified
2	Sakhelashvili et al.	Bdq, Dlm	–	–	–	–	–	–	–	–	–	–
3	Mohr-Holland et al.	Bdq / Dlm / Bdq + Dlm	4/22 (18%)	4/22 (18%)	–	–	–	–	–	–	1/22 (4.5%)	Linezolid
4	Das et al.	Bdq / Dlm / Bdq + Dlm (background regimen)	–	–	–	–	–	–	–	–	–	–
5	Achar et al.	Bdq, Lzd, Cfz, Mfx, Imi	5/20 (25%)	–	–	–	–	–	–	–	–	Bedaquiline
6	Pirmahmadzoda et al.	Bdq, Dlm, Cm/Am, Lfx/Mfx, Pto, Cs, PAS, Lzd, Cfz	–	–	–	–	–	–	–	–	–	–
7	Ghosh et al.	Dlm + background regimen	–	–	–	–	–	–	–	–	–	–

8	Hafkin et al.	Bdq, Dlm (combined)	–	–	–	–	–	–	–	–	–	–
9	Moodliar et al.	Bdq + background regimen	12/30 (40%)	–	–	1/30 (3.3%)	2/30 (6.7%)	–	–	–	–	Not specified
10	Garcia-Prats et al.	Dlm + optimized background regimen	2/37 (5.4%)	–	–	–	–	–	1/37 (2.7%)	1/37 (2.7%)	–	Delamanid plus OBR
11	Smirnova et al.	WHO-based regimen (not specified)	10/52 (19.2%)	–	5/52 (9.6%)	–	–	5/52 (9.6%)	–	–	–	Not specified
12	Da Costa et al.	Cm, EMB, PZA, Lfx, Trd	–	–	–	–	–	–	–	–	–	–
13	Sun et al.	Bdq, FQ, Lzd, Cs, Cfz, PZA, Pto, PAS, EMB	13/79 (16.5%)	–	–	–	–	–	–	–	–	Not specified
14	Mesic et al.	Bdq, Dlm, Lfx, Lzd, Cfz, Z, Cs	–	–	–	–	–	–	–	–	–	–

The table summarizes adverse events reported as either serious (according to ICH/FDA criteria) or as Grade 3–4 (based on the Common Terminology Criteria for Adverse Events, CTCAE). Some studies reported the total number of such events without detailing individual conditions. Therefore, the columns do not represent all side effects observed but only those for which individual breakdowns were provided. OBR = Optimized Background Regimen. Bdq = bedaquiline; Dlm = delamanid; Lzd = linezolid; Cfz = clofazimine; FQ = fluoroquinolone; Mfx = moxifloxacin; Lfx = levofloxacin; Imi = imipenem; Cs = cycloserine; Pto = protionamide; PAS = para-aminosalicylic acid; Cm = capreomycin; Am = amikacin; EMB = ethambutol; PZA or Z = pyrazinamide; Trd = terizidone.

SUPPLEMENTAL DIGITAL CONTENT 12. Quality assessments of observational studies using Newcastle-Ottawa Scale

Study (year)	A. Selection (maximum of four stars)				B. Comparability (maximum of two stars)	C. Outcome (maximum of three stars)			Total (maximum of nine stars)
	1. Representativeness of the exposed cohort	2. Selection of the non-exposed cohort	3. Ascertainment of exposure	4. Demonstration that outcome of interest was not present at start of study	1. Comparability of cohort on the basis of the design or analysis	1. Assessment of outcome	2. Was follow-up long enough for outcomes to occur	3. Adequacy of follow-up of cohorts	
Solodovnikova et al. (2021)	★	☆	★	★	★	★	★	★	7
Sakhelashvili et al (2023)	★	★	★	★	★	★	★	★	8
Mohr-Holland et al (2020)	★	☆	★	★	★	★	★	★	7
Das et al (2020)	★	☆	★	★	★	★	★	★	7
Achar et al (2017)	★	☆	★	★	★	★	★	★	7
Pirmahmadzoda (2021)	★	☆	★	★	★	★	★	★	7
Ghosh et al (2022)	★	☆	★	★	★★	★	★	★	8

**Study (year)**	**A. Selection (maximum of four stars)**				**B. Comparability (maximum of two stars)**	**C. Outcome (maximum of three stars)**			**Total (maximum of nine stars)**
	1. Representativeness of the exposed cohort	2. Selection of the non-exposed cohort	3. Ascertainment of exposure	4. Demonstration that outcome of interest was not present at start of study	1. Comparability of cohort on the basis of the design or analysis	1. Assessment of outcome	2. Was follow-up long enough for outcomes to occur	3. Adequacy of follow-up of cohorts	
Hafkin et al (2018)	★	☆	★	★	★	★	★	★	7
Smirnova et al (2016)	★	☆	★	★	★	★	★	★	7
Da Costa et al. (2023)	★	☆	★	★	★	★	★	★	7
Sun et al. (2024)	★	★	★	★	★★	★	★	★	9

Note:

Total Score (Out of 9)	Quality Rating
7-9 stars	Good Quality
5-6 stars	Fair Quality
0-4 stars	Poor Quality

SUPPLEMENTAL DIGITAL CONTENT 13. Risk bias assessment for RCTs using Cochrane tool

	Risk of bias domains					
	D1	D2	D3	D4	D5	Overall
Garcia-Prats et al (2022)	!	!	+	+	+	!
Moodliar et al (2021)	-	+	+	+	+	!
Mesic et al (2024)	!	+	+	+	+	!

Domains

D1: Bias arising from the randomization process
D2: Bias due to deviations from intended interventions
D3: Bias due to missing outcome data
D4: Bias in measurement of the outcome
D5: Bias in selection of the reported result

Judgement

+ Low risk
! Some concerns
- High risk

SUPPLEMENTAL DIGITAL CONTENT 14. Funnel plot (on overall outcome)

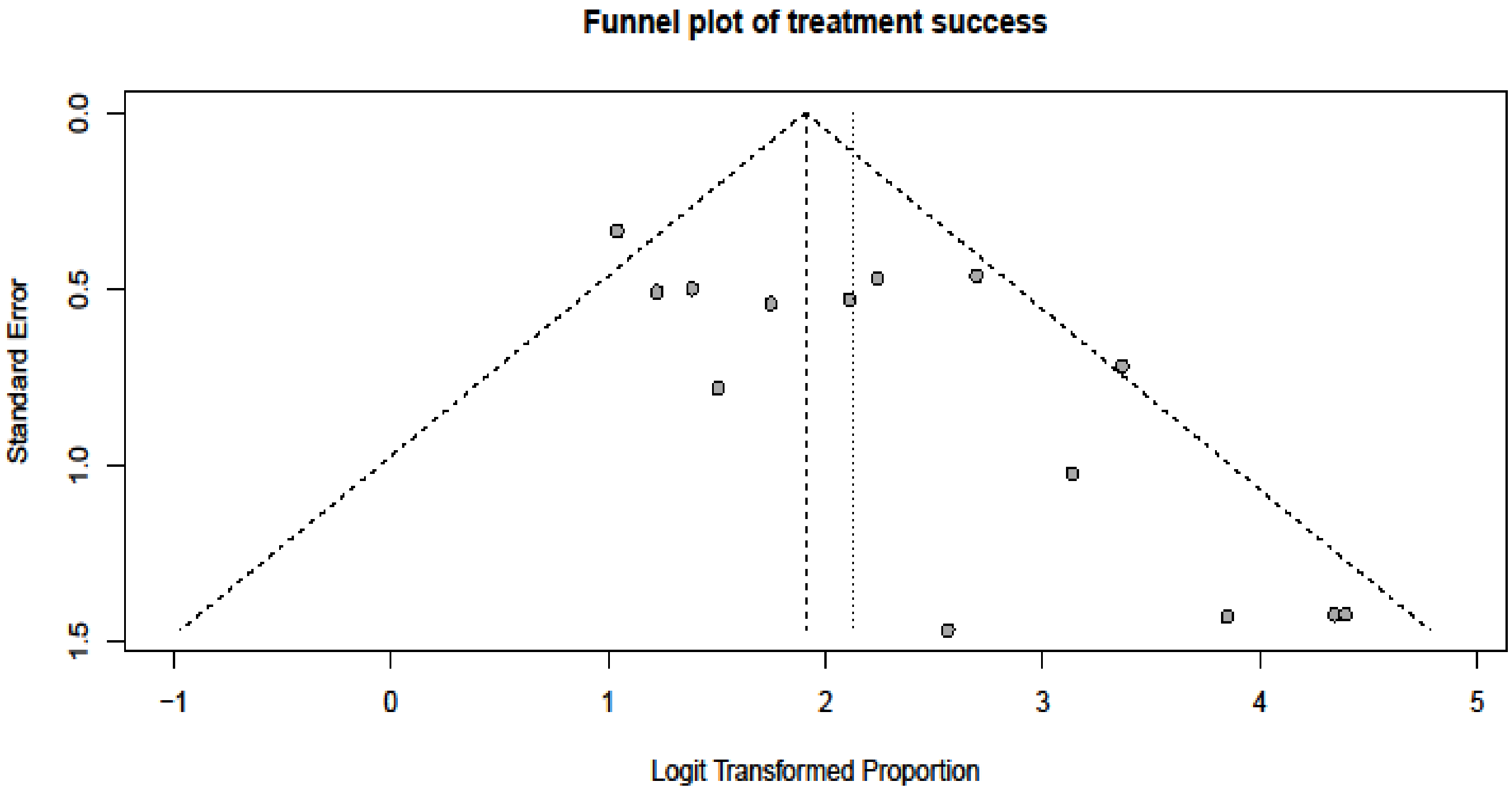

SUPPLEMENTAL DIGITAL CONTENT 15. Egger's test (on overall outcome)

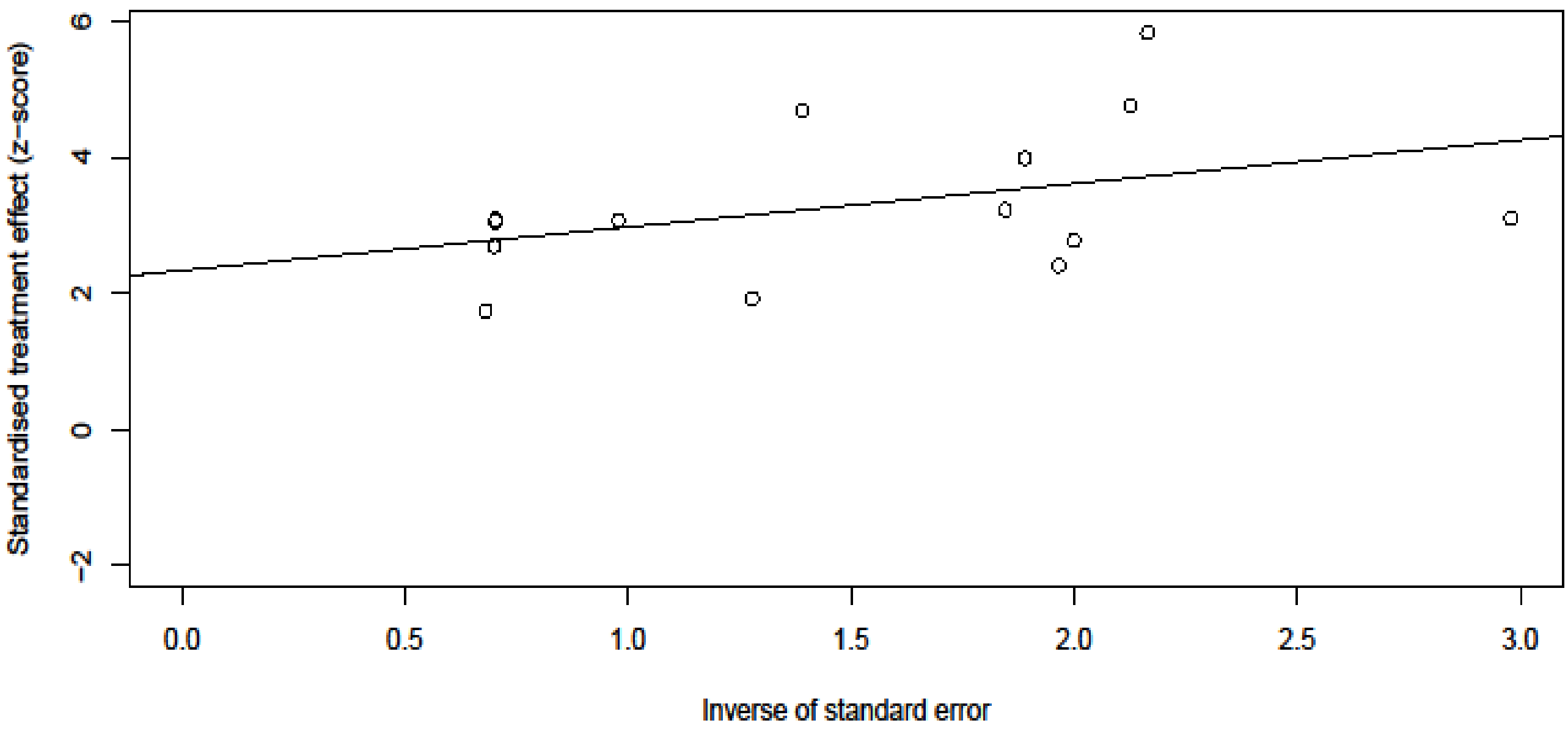


```
Linear regression test of funnel plot asymmetry

Test result: t = 3.30, df = 12, p-value = 0.0063
Bias estimate: 2.3331 (SE = 0.7061)
```

SUPPLEMENTAL DIGITAL CONTENT 16. Summary of findings:

Effectiveness and safety of contemporary drug regimens for multidrug- and extensively drug-resistant tuberculosis compared to none in children and adolescents

Patient or population: children and adolescents

Setting:

Intervention: effectiveness and safety of contemporary drug regimens for multidrug- and extensively drug-resistant tuberculosis

Comparison: none

Outcomes	Absolute effect (95% CI)	№ of participants (studies)	Certainty of the evidence (GRADE)	Comments
Treatment success assessed with: Proportion	**0.89** (0.84 to 0.93)	490 (14 non-randomised studies)	⨁◯◯◯ Very low[a,b]	The evidence is very uncertain about the effect of effectiveness and safety of contemporary drug regimens for multidrug- and extensively drug-resistant tuberculosis on treatment success.

CI: confidence interval

GRADE Working Group grades of evidence
High certainty: we are very confident that the true effect lies close to that of the estimate of the effect.
Moderate certainty: we are moderately confident in the effect estimate: the true effect is likely to be close to the estimate of the effect, but there is a possibility that it is substantially different.
Low certainty: our confidence in the effect estimate is limited: the true effect may be substantially different from the estimate of the effect.
Very low certainty: we have very little confidence in the effect estimate: the true effect is likely to be substantially different from the estimate of effect.

Explanations

a. Non randomised studdies

b. Heterogenicity was moderate
